# Sex and age significantly modulate cardiovascular disease presentation in type 2 diabetes: a large population-based cohort study

**DOI:** 10.3389/fendo.2024.1344007

**Published:** 2024-05-17

**Authors:** Amanda Jiménez, Bogdan Vlacho, Manel Mata-Cases, Jordi Real, Dídac Mauricio, Josep Franch-Nadal, Emilio Ortega

**Affiliations:** ^1^ Department of Endocrinology & Nutrition, Hospital Clinic Barcelona, Barcelona, Spain; ^2^ CIBER of Obesity and Nutrition (CIBEROBN), Instituto de Salud Carlos III (ISCIII), Barcelona, Spain; ^3^ Instituto de Investigaciones Biomédicas August Pi i Sunyer (IDIBAPS), Barcelona, Spain; ^4^ DAP-Cat group, Unitat de Suport a la Recerca Barcelona, Fundació Institut Universitari per a la recerca a l’Atenció Primària de Salut Jordi Gol i Gurina (IDIAPJGol), Barcelona, Spain; ^5^ Centro de Investigación Biomédica en Red (CIBER) of Diabetes and Associated Metabolic Diseases (CIBERDEM), Instituto de Salud Carlos III (ISCIII), Barcelona, Spain; ^6^ Department of Endocrinology & Nutrition, Hospital de la Santa Creu i Sant Pau, Barcelona, Spain; ^7^ Departament of Medicine, University of Vic - Central University of Catalonia, Vic, Spain; ^8^ Primary Health Care Center Raval Sud, Gerència d’Atenció Primària Barcelona Ciutat, Institut Català de la Salut, Barcelona, Spain

**Keywords:** first cardiovascular event, primary prevention, heart failure, peripheral artery disease, sex difference, age-related, cumulative incidence

## Abstract

**Aims:**

We aimed to describe and compare the incidence of the first cardiovascular event and its major subtypes, coronary heart disease (CHD), cerebrovascular disease, heart failure (HF), or peripheral artery disease (PAD), according to age and sex in a population-based cohort of individuals with type 2 diabetes (T2D) from a Mediterranean region.

**Material and methods:**

We used linked primary care electronic medical reports, pharmacy-invoicing data, and hospital admission disease registry records from the SIDIAP database, which contains linked data for 74% of the Catalonian population. We selected individuals with T2D aged 30 to 89 years free of cardiovascular disease (CVD). The primary outcome was the first presentation of CVD.

**Results:**

The study cohort included 247,751 individuals (48.6% women, 66.8 ± 11.9 years). During a 6.99-year follow-up, the cumulative incidence of the first cardiovascular event was 23.4%. Men were at higher risk for CVD (hazard ratio [HR]: 1.47 95%CI: 1.45-1.50), CHD (HR: 1.52 95%CI: 1.47-1.57), cerebrovascular disease (HR:1.07 95%CI: 1.03-1.10) and PAD (HR: 2.30 95%CI: 2.21-2.39) than women but at a lower risk for HF (HR:0.70 95%CI: 0.68-0.73). CHD and PAD were the most frequent CVD presentations among men (28.1% and 27.5%) and HF (40.1%) in women. CHD predominated among young participants of both sexes, while HF predominated among women older than 65 and men older than 75.

**Conclusions:**

In individuals with T2D, the overall risk and the type of first CVD manifestation largely varied by sex and age. This epidemiological evidence should be considered in clinical practice.

## 1 Introduction

Patients with type 2 diabetes (T2D) are at a higher risk for a wide range of cardiovascular diseases (1). Although a substantial reduction in incidence rates of cardiovascular complications has been observed over the last two decades, cardiovascular disease (CVD) risk in T2D remains higher than in age and sex-matched controls. Furthermore, despite steadily decreasing rates for atherosclerotic diseases, the decline in heart failure (HF) incidence has plateaued in recent years (2).

Given the varied impact of modifiable cardiovascular risk factors on different manifestations of CVD, acquiring a comprehensive understanding of the epidemiology surrounding initial cardiovascular events in individuals with T2D could foster the creation of more personalized primary prevention approaches (2). This might be especially relevant among young adults, as evidenced by recent data from the Global Burden of Disease (GBD) study, which highlights an increasing trend in cardiovascular morbidity and mortality in this age cohort over the last three decades (3).

In this context, there is a growing acknowledgment that CVD pathophysiology and presentation exhibit variations based on sex and age (4). Of note, within the general population, CVD predominantly presents as coronary heart disease (CHD) in men, whereas women are more prone to experiencing cerebrovascular disease or HF as their primary event, with these manifestations typically occurring more frequently at older ages (5). Whether such age and sex differences in the first CVD event are also present in individuals with T2D has seldom been explored.

Several studies and meta-analyses have demonstrated a stronger association between T2D and HF, CHD, and cerebrovascular disease in women than in men (6–11). Fewer studies have explored sex differences in absolute risk for the various forms of CVD manifestation, and none focused on first events (2, 6, 12). In addition, whether age modulates sex-specific risks for the different CVD subtypes in men and women with T2D is poorly defined as most previous studies applied broad age groups or did not consider the various CVD components separately (2, 6, 12).

In this work, we aimed to describe and compare the incidence rates of the first cardiovascular event and its major subtypes (CHD, PAD, cerebrovascular disease, and HF) according to sex and age in a large cohort of individuals with T2D from a primary care setting.

## 2 Materials and methods

### 2.1 Study design and settings

We performed a longitudinal, retrospective cohort study. Data for this study was obtained from the Sistema d’Informació per al Desenvolupament de l’Investigació en Atenció Primària (SIDIAP) database (www.sidiap.org). This database, created in 2010 for research purposes, contains pseudo-anonymous, routinely collected healthcare information of over 5,000,000 patients registered by 3,414 general practitioners at 274 different primary care practices in Catalonia (a northeast area of Spain). The population in the SIDIAP database corresponds to more than 74% of the Catalonian population, providing a representative sample of the primary care population.

The SIDIAP database contains information on clinical diagnoses, anthropometric measures, and laboratory tests. In addition, the CMBD database (data from hospitalisation discharge statistics and specialised out-patient care from the hospitals of the National Health System) and pharmacological treatments (data from the pharmacy-invoicing system provided by the CatSalut) are automatically collected and linked to the SIDIAP database. The Ethics Committee of the Primary Healthcare University Research Institute (IDIAP), Jordi Gol (Barcelona, Spain), approved the study (code P17/087).

### 2.2 Study population

For this analysis, we selected all individuals with a registered diagnosis of T2D (n=333,036) on January 1st, 2010 (study index date). T2D diagnosis was based on the presence of the diagnostic codes (International Statistical Classification of Diseases and Related Health Problems 10th Revision-ICD-10): E11, E13, and E14. Subjects with other types of diabetes, such as type 1, secondary, or gestational diabetes (ICD-10: E10, E12, O24), were excluded from the analysis.

Individuals with previous cardiovascular events (n=68,982, 20.7%) or known atrial fibrillation (n=22,673, 6.8%) were excluded, as well as those aged under 27 or above 89 years (n=8,310, 2.4%) for whom cardiovascular risk evaluation was not systematically recommended or CVD prevention of a first event was not a clinical priority. The remaining participants (n=247,751) were followed until the cardiovascular event, death, or the end of the study (December 2016). Study flow charts are included in the **Supplementary material** (**Supplementary Figure 1**).

The study population was divided into five different age groups according to baseline age and sex (young (Y): <35 years; early adulthood (EA): 35-55 for men and 35-60 for women; middle adulthood (MA): 55-65 for men and 60-65 for women; young old (YO): 65-75 years; middle-to-very old (MVO): >75 years). This age grouping was selected considering the age range at which systematic cardiovascular risk evaluation is recommended (Framingham-REGICOR strategy 35-75 years) in our Health Care System, and also according to the age limit to define premature events according to sex, i.e., <55 years for men and <60 years for women (13).

### 2.3 Study variables

At the study index date, the following data were collected from the SIDIAP database: age, sex, socioeconomic condition, the presence of diagnoses and comorbidities, including microvascular complications (retinopathy, nephropathy, polyneuropathy), and hypertension (based on registers of ICD-9/ICD-10 diagnostic codes recorded in the database) use of concomitant medications (based on the Anatomical Therapeutic Chemical-ATC classification system), blood pressure, anthropometric (body weight, height, and body mass index) and laboratory data [lipids, creatinine, urinary albumin excretion (UAE)].

Specific diagnosis codes for retinopathy, nephropathy, polyneuropathy, and hypertension are displayed in **Supplementary Table 1**.

Any microvascular complication was defined as the presence of a diagnostic code of retinopathy or/and nephropathy or/and polyneuropathy. Obesity was described as a body mass index ≥30 Kg/m2. The socioeconomic condition was evaluated by the MEDEA index. The MEDEA index is a socioeconomic deprivation index based on indicators recorded by census tract and validated in the Spanish population. Higher index values correspond to greater deprivation (14). The estimated glomerular filtration rate (eGFR) was calculated using the Chronic Kidney Disease Epidemiology Collaboration (CKD-EPI) equation.

### 2.4 Study outcomes

Cardiovascular events involving four major conditions occurring after the index date and before the end of the study period were recorded and reported as CHD, cerebrovascular disease, PAD, and HF. CHD was defined as fatal or non-fatal myocardial infarction, angina or unstable angina, undetermined ischemic heart disease, and coronary revascularisation (coronary artery bypass grafting, percutaneous coronary intervention). Cerebrovascular disease was defined as fatal or non-fatal ischemic or hemorrhagic stroke, transient ischemic attack, and intracerebral revascularisation. PAD was defined as intermittent claudication, extracerebral artery stenosis, and carotid or peripheral revascularisation (endovascular, stenting, or surgical bypass). HF included congestive/acute HF and other HF diagnoses (systolic and diastolic, chronic, or undetermined). Diagnoses were based on ICD-9/ICD-10 codes. Specific diagnosis codes for CVD can be found in the online **Supplementary information** (**Supplementary Table 2**).

The overall mortality during the study period was also retrieved from the SIDIAP database. However, specific causes of death were not available in this study.

### 2.5 Statistical methods

The baseline characteristics were described as frequencies and percentages for categorical variables, while for continuous variables, the mean and standard deviation (SD) or median and quartiles were calculated. In comparing groups (sex), the p-value was calculated using the Fisher exact test for qualitative variables and the independent samples t-test for quantitative variables. Fisher exact test and ANOVA analysis were applied when comparing age categories.

Cumulative incidence and incidence rates of cardiovascular manifestations were computed using the exact method. For each event of interest, the overall incidence rate and age-sex-specific incidence rate were calculated as the number of incident events divided by the persons-years (PY) during the follow-up and were expressed as per 100 PY. Hazard ratios (HR) and 95% confidence intervals (95% CI) were computed for each baseline characteristic. The *compare Group*s R Package (Version 4.6.0) 10 was used to perform group descriptions for several variables and to estimate each HR. Cox proportional hazard models were used to evaluate time to death according to the type of first-ever cardiovascular disease presentation. Hazard ratios (HR) in these models were adjusted by age and sex.

All statistical analyses were performed using the free R statistical software version 3.6.1 (https://www.r-project.org/).

## 3 Results

The study cohort included 247,751 individuals (48.6% women) with T2D. **Table 1** displays the clinical characteristics at baseline for the whole cohort and stratified by age.

**Table 1:** Clinical characteristics at baseline for the whole cohort and stratified by age.

	% with Available data	Whole cohort (n=247,751)	Young (n=1,204)	Early adulthood (n=51,295)	Middle Adulthood (n=52,636)	Young old (n=72,655)	Middle to very old (n=69,961)	p_trend_
**Females (n, (%))**	100.0	120,405 (48.6)	540 (44.9)	25,399 (49.5)	15,907 (30.2)	36,113 (49.7)	42,446 (60.7)	<0.01
**Age (years)**	100.0	66.8 ± 11.9	32.9 ± 1.38	50.4 ± 5.75	61.0 ± 2.71	69.9 ± 2.95	80.7 ± 3.91	<0.01
**Deprivation index (Q5)** **(n, (%))**	70.0	29,521 (17.0)	88 (10.2)	5,080 (13.6)	65,45 (17.0)	8,624 (16.4)	9,184 (20.8)	<0.01
Smoking (n, (%)								
No	86.5	137,190 (64.0)	483 (50.9)	21,723 (49.5)	23,778 (49.5)	43,809 (68.4)	47,397 (79.8)	<0.01
Former		39,370 (18.4)	359 (37.8)	15,025 (34.2)	11,932 (25.5)	8,362 (13.0)	3,692 (6.2)	
Current		37,622 (17.6)	107 (11.3)	7,157 (16.3)	10,223 (23.3)	11,847 (17.5)	8,288 (14.0)	
**T2D duration (years)**	100.0	6.3 ± 5.2	3.6 ± 3.3	4.7 ± 4.0	5.6 ± 4.5	6.7 ± 5.2	7.6 ± 6.1	<0.01
T2D therapy (n, (%))								
No treatment	100.0	66,806 (27.0)	527 (43.8)	15,139 (29.5)	13,827 (26.3)	18,204 (25.1)	19,109 (27.3)	<0.01
OHA monotherapy		84,419 (34.1)	359 (29.8)	18,059 (35.2)	18,496 (35.1)	24,790 (34.1)	22,715 (32.5)	
OHA combined		57,469 (23.2)	139 (11.5)	10,565 (20.6)	12,883 (24.5)	18,106 (24.9)	15,776 (22.5)	
OHA plus insulin		2,6015 (10.5)	100 (8.3)	5,113 (10.0)	5,180 (9.8)	8,212 (11.3)	7,410 (10.6)	
Insulin without OHA		13,042 (5.3)	79 (6.6)	2,419 (4.7)	2,250 (4.3)	3,343 (4.6)	4,951 (7.1)	
Use of non-insulin hypoglycemic medications (n, (%))								
Metformin	100.0	146,793 (59.3)	565 (46.9)	31,380 (61.2)	33,292 (63.2)	44,842 (61.7)	36,714 (52.5)	<0.01
Sulfonylureas		72,955 (29.4)	132 (11.0)	11,677 (22.8)	14,910 (28.3)	22,862 (31.5)	23,374 (33.4)	<0.01
DPP-4 inhibitors		13,080 (5.3)	48 (4.0)	2,950 (5.8%)	3,207 (6.1%)	4,114 (5.7)	2,761 (3.9)	<0.01
SGLT2-inhibitors		0 (0.0)	0 (0.0)	0 (0.0)	0 (0.0)	0 (0.0)	0 (0.0)	<0.01
GLP1-analogs		722 (0.3)	9 (0.7)	353 (0.7)	198 (0.4)	142 (0.2)	20 (0.0)	<0.01
Pioglitazone		8,954 (3.6)	33 (2.7)	1,927 (3.8)	2,115 (4.0)	2,930 (4.0)	1,949 (2.8)	<0.01
Glinides		4,406 (1.8)	3 (0.2)	377 (0.7)	677 (1.3)	1,426 (2.0)	1.923 (2.7)	<0.01
**FPG (mg/dl)**	70.1	146 ± 46.6	149 ± 64.6	155 ± 55.5	151 ± 47.8	144 ± 42.2	137 ± 41.6	<0.01
**HbA1c (%)**	65.6	6.8 ± 1.5	7.1 ± 2.1	7.1 ± 1.8	6.9 ± 1.6	6.7 ± 1.4	6.7 ± 1.3	
**BMI (Kg/m^2^)**	54.9	30.2 ± 5.1	31.9 ± 7.0	31.6 ± 5.9	30.5 ± 4.9	30.2 ± 4.8	29.1 ± 4.6	<0.01
**BMI≥30 Kg/m^2^** **(n, (%))**	54.9	61,632 (46.3)	280 (57.0)	14,137 (56.4)	13,524 (48.3)	19,745 (45.9)	13,910 (38.1)	<0.01
**Hypertension (n (%))**	100.0	156,967 (63.4)	177 (14.7)	22,108 (43.1)	30,635 (58.2)	50,770 (69.9)	53,277 (76.2)	<0.01
**SBP (mmHg)**	75.5	136 ± 15.6	127 ± 14.1	132 ± 15.2	136 ± 15.2	137 ± 15.4)	138 ± 16.1	<0.01
**DBP (mmHg)**	75.5	76.8 ± 9.4	78.6 ± 10.6	80.4 ± 9.4	78.9 ± 9.0	76.3 ± 8.9	73.6 ± 9.2	<0.01
**Hypotensive treatment (n (%))**	100.0	162,424 (65.6)	181 (15.0)	23,118 (45.1)	32,147 (61.1)	52,729 (72.6)	54,249 (77.5)	<0.01
**Total cholesterol (mg/dl)**	68.7	197 ± 38.8	196 ± 50.1	206 ± 42.4	199 ± 39.2	195 ± 36.8	193 ± 37.0	<0.01
**HDL-cholesterol (mg/dl)**	60.1	50.2 ± 13.0	43.9 ± 12.8	47.5 ± 12.6	48.8 ± 12.6	51.0 ± 12.7	52.3 ± 13.5	<0.01
**Triglycerides (mg/dl)**	64.8	133.0 [97.0-186]	157.0 [103-235]	152.0 [107-224]	139.0 [100-197]	129.0 [96.0-177]	122.0 [92.0-165]	<0.01
**LDL-cholesterol (mg/dl)**	60.1	118 ± 32.8	117 ± 32.9	124 ± 34.7	120 ± 33.4	116 ± 31.7	114 ± 31.4	<0.01
**Lipid-lowering treatment (n,(%))**	100.0	123,901 (50.0)	194 (16.1)	21,117 (41.2)	27,402 (52.1)	41,141 (56.6)	34,047 (48.7)	<0.01
**Statin use (n,(%))**	100.0	112,905 (45.6)	134 (11.1)	17,825 (34.8)	24,557 (46.7)	38,361 (52.8)	32,028 (45.8)	<0.01
**Fibrates use (n,(%))**	100.0	15,647 (6.3)	76 (6.3)	4,563 (8.9)	4,212 (8.0)	4,133 (5.7)	2,663 (3.8)	<0.01
eGFR categories (n, (%))								
>60 ml/min	67.2	136,906 (82.2)	575 (99.5)	31,007 (97.5)	32,796 (93.7)	43,239 (83.9)	29,289 (61.6)	<0.01
30-60 ml/min		27,662 (16.6)	2 (0.3)	728 (2.3)	2,070 (5.9)	7,904 (15.3)	16,958 (35.7)	
<30 ml/min		1,874 (1.1)	1 (0.2)	61 (0.2)	121 (0.3)	393 (0.8)	1,298 (2.7)	
**Diabetic retinopathy** **(n (%))**	100.0	11,041 (4.5)	9 (0.7)	1,488 (2.9)	2,361 (4.5)	3,623 (5.0)	3,560 (5.1)	<0.01
**Diabetic nephropathy (n (%))**	100.0	15,645 (6.3)	24 (2.0)	1,684 (3.3)	2,310 (4.4)	4,402 (6.1)	7225 (10.3)	<0.01
**Diabetic neuropathy (n (%))**	100.0	4,278 (1.7)	2 (0.2)	594 (1.2)	858 (1.6)	1,358 (1.9)	1,466 (2.1)	<0.01
**Any microvascular complication (n (%))**	100.0	27,846 (11.2)	31 (2.6)	3,392 (6.6)	4,928 (9.4)	8,373 (11.5)	11,122 (15.9)	<0.01

Data are expressed as mean ± standard deviation, median [25^th^ percentile-75^th^ percentile], or number of participants (n) (%).

OHA, oral hypoglycemic agents; FPG, fasting plasma glucose; BMI, body mass index; SBP, systolic blood pressure; DBP, diastolic blood pressure; HDL-cholesterol, high-density lipoprotein cholesterol; LDL-cholesterol, low-density lipoprotein cholesterol; eGFR, estimated glomerular filtration rate.

The mean age was 66.8 ± 11.9 years, HbA1c was 6.8 ± 1.5%, and body mass index (BMI) was 30.2 ± 5.1 Kg/m^2^. The most common treatment modalities were oral hypoglycemic agents in monotherapy or combined. Metformin (59.3%) and sulfonylureas (29.5%) were the most frequently prescribed hypoglycemic medications. In contrast, a mere 0.3% of participants utilized glucagon-like peptide 1 analogs, and none were prescribed sodium glucose transporter inhibitors (SGLT2-i). The prevalence of hypertension, statin use, current smoking, and microvascular complications was 63.4%, 45.6%, 17.6%, and 11.2%, respectively.

Compared to the older age groups, the younger groups had a higher proportion of males, a shorter diabetes duration, were less often treated with insulin, and had a lower prevalence of hypertension and microvascular complications. In contrast, they showed worse glycemic control and lipid profile, had a higher prevalence of obesity, and were less frequently treated with lipid-lowering therapies. The prevalence of active smokers was the lowest among the young, increasing until middle adulthood and decreasing after that (**Table 1**).

### 3.1 Incidence of first cardiovascular event stratified by age and sex

**Figure 1:**
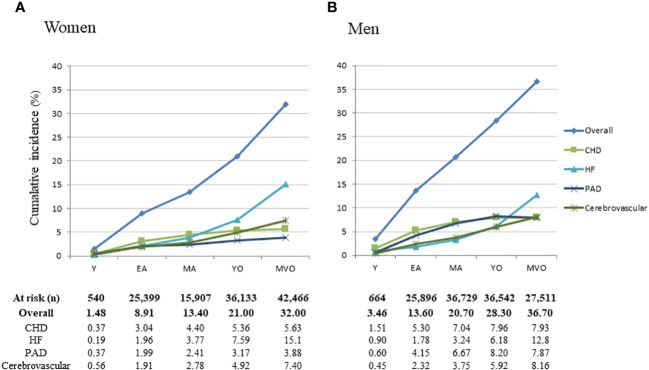
Cumulative incidence rates for first cardiovascular events in women **(A)** and men **(B)** by age categories. CVD, cardiovascular disease; CHD, coronary heart disease; HF, heart failure; PAD, peripheral artery disease; Y, young; EA, early adulthood; MA, middle-adulthood; YO, young old; MVO, middle to very old.

The cumulative incidence of overall CVD and its major components in men and women according to age category is displayed in **Figure 1**.

During 1,435,568 person-years, first-time cardiovascular events occurred in 57,152 individuals, corresponding to a cumulative incidence of 21.2% in women and 24.8% in men and a crude incidence rate of 3.61% per year in women and 4.34% in men (p<0.01).

The incidence of cardiovascular events increased across age categories in both men and women (p_trend_<0.01), with a maximum incidence among middle to very-old adults (HR: 4.03, 95%CI: 3.91-4.16, with young as the reference). Men were at a higher absolute risk than women. The excess risk for men was the highest in young adults (HR: 2.38 (95%CI: 1.06 to 5.32) and the lowest among middle to very-old adults (HR: 1.25; 95%CI: 1.22 to 1.28).

**Figure 2:**
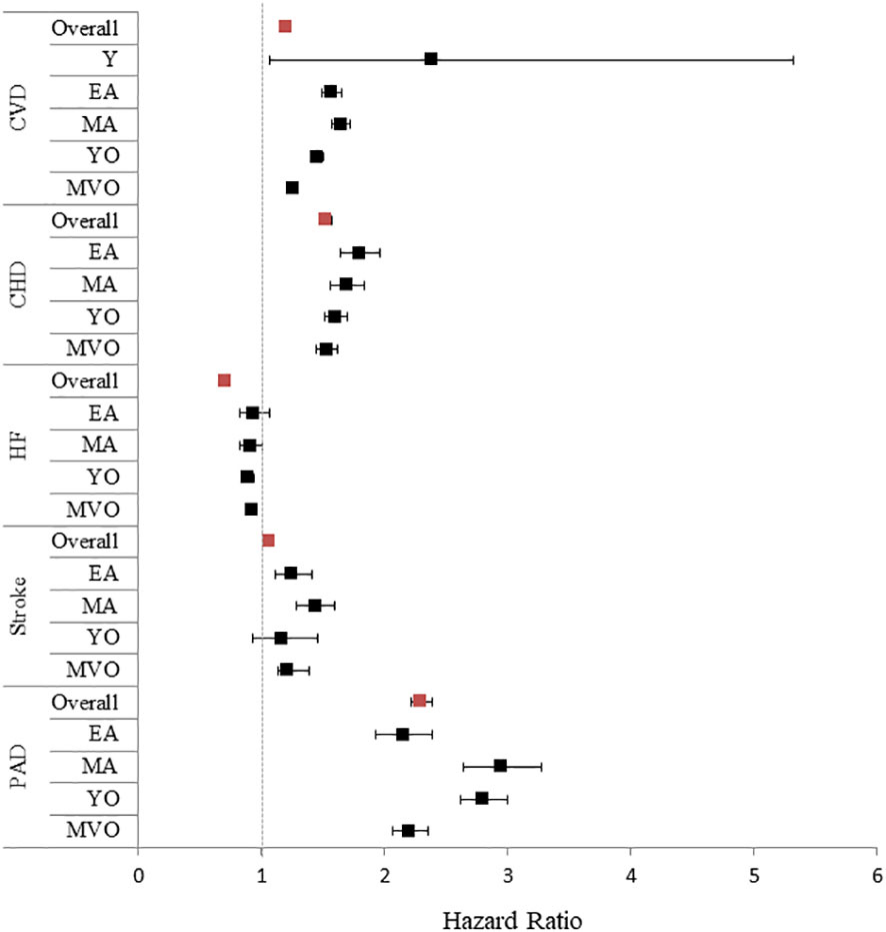
Forrest plot for a first cardiovascular event stratified by age in women vs. men Women were considered as the reference. Given the low number of events among the young age category, the hazard ratios for cardiovascular disease subtypes were not included in the figure. Red squares: whole cohort; black squares: pre-defined age subgroups. Y, young; EA, early adulthood; MA, middle-adulthood; YO, young old; MVO, middle to very old. CVD, cardiovascular disease; CHD, coronary heart disease; HF, heart failure; PAD, peripheral artery disease.

When different CVD subtypes were considered individually, men were at higher risk than women for CHD, PAD, and cerebrovascular disease (p<0.01). In contrast, women showed a higher risk than men for HF (p<0.01) (**Figures 1**, **2**).

### 3.2 The first cardiovascular event reported

**Figure 3:**
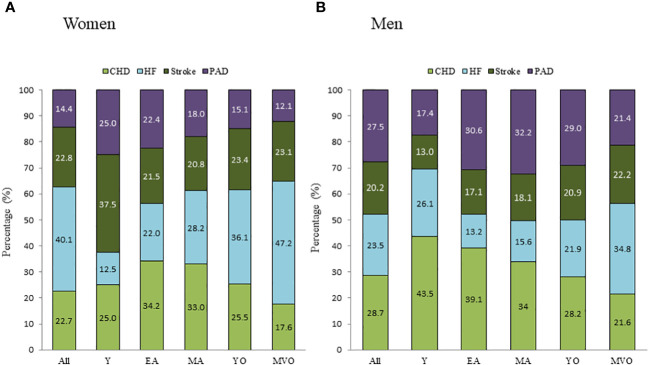
Distribution of the first-ever cardiovascular event subtype stratified by sex in the different age categories. **(A)**: women; **(B)**: men. Y, young; EA, early adulthood; MA, middle-adulthood; YO, young old; MVO, middle to very old; CVD, cardiovascular disease; CHD, coronary heart disease; HF, heart failure; PAD, peripheral artery disease.

**Figure 3**; **Supplementary Figure 2** display the relative contribution of cardiovascular disease main categories and specific subtypes to the first CVD event in men and women for the entire cohort and stratified by age.

In men, the most frequent CVD presentation was CHD (28.7%), followed by PAD (27.5%), while in women, HF clearly predominated (40.1%).

Furthermore, the first CVD event differed according to age in both men and women. Although more frequent among men, CHD was the most frequent first CVD manifestation in both men and women during early and middle adulthood, decreasing after that and becoming less common in older groups. Despite this reducing incidence with age, CHD remained the most frequent presentation of CVD among men below 75 years and women below 65 years. Similar trends were observed for PAD, which was much more common among men than women and more frequent among younger than older groups.

The opposite was observed for HF, which, although more common in women than men at any age, was relatively infrequent in younger age categories but markedly increased in the older groups. In women, the contribution of HF to overall CVD doubled between EA and MVO (22.0% to 47.1%), while in men, it tripled (13.3% to 34.8%), representing the most common presentation of CVD in the YO and MVO groups among women (i.e., >65 years), and the MVO group among men (i.e., >75 years). The proportion of cerebrovascular disease as the first cardiovascular event varied less with age and between sexes (18.7% to 22.7%) (**Figure 3**).

### 3.3 Factors related to cardiovascular events stratified by age and sex

The baseline characteristics of individuals with or without cardiovascular events during the follow-up are shown in **Supplementary Table 3**.

In sex-stratified analyses adjusted by age, most CVD risk factors were significantly and similarly associated with CVD in both men and women. However, compared to men, women had a higher negative impact of deprivation [HR: 1.24 (95%CI: 1.17-1.30) vs. HR: 1.12 (95%CI: 1.07-1.17)], obesity [(HR: 1.31 (95%CI: 1.26-1.35) vs. HR: 1.13 (95%CI: 1.10-1.17)], and hypertension (HR: 1.43 (95%CI: 1.38-1.47) vs. HR: 1.27 (95%CI: 1.24-1.30)] (**Supplementary Figure 3**), Factors more strongly associated with CVD in these analyses were those related to T2D duration, control, and complications.

In age-stratified analyses, we observed a higher relative risk associated with classical cardiovascular risk factors, metabolic control, and end-organ damage in younger vs. older individuals (**Supplementary Figure 4**).

### 3.4 Mortality during follow-up

During the follow-up, 20,811 men (16.3%) and 18,866 women (15.7%) died. The mean age at the time of death was 73.7 ± 10.2 years in men and 78.4 ± 8.5 years in women. Before the death date, 52.7% of deceased men and 47.9% of dead women had had a previous first cardiovascular event. The specific cause of death was not available.

**Figure 4:**
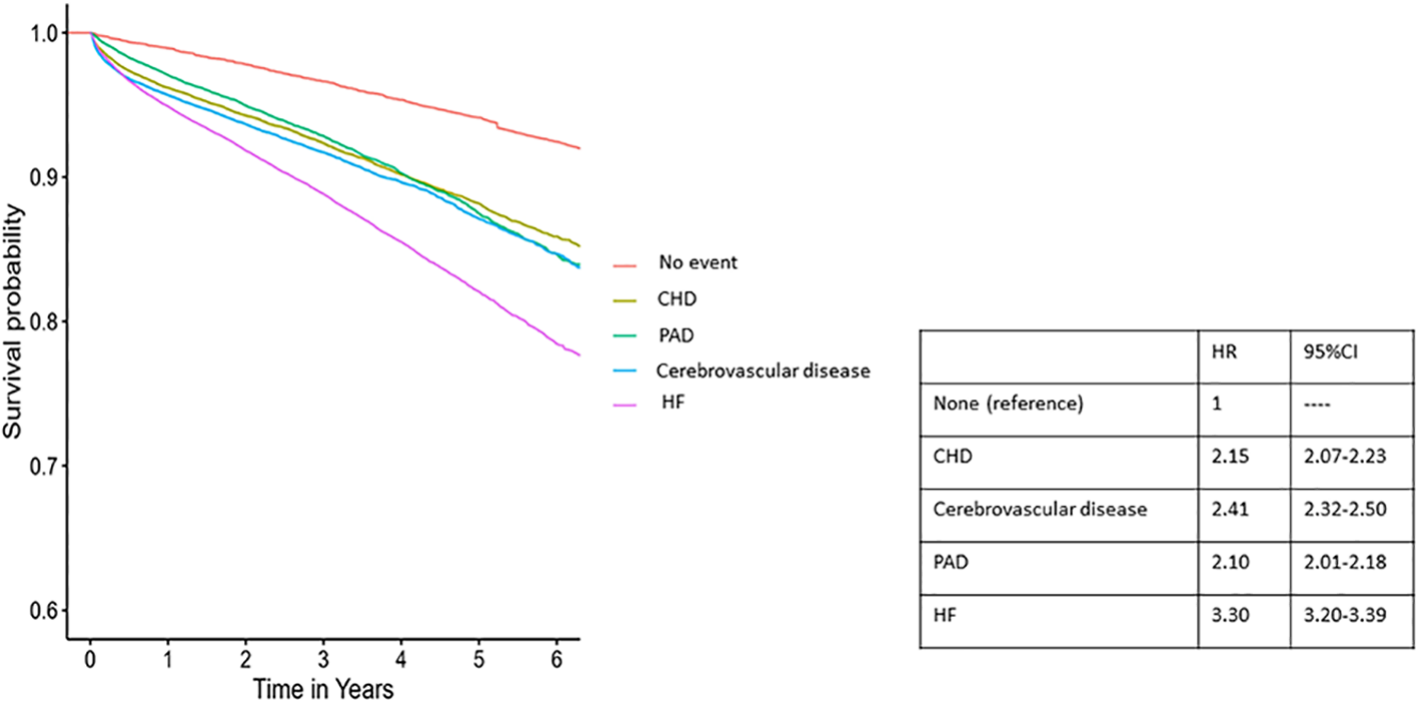
Survival plot for all-cause mortality according to first cardiovascular event form of manifestation and the corresponding hazard ratios CHD, coronary heart disease; HF, heart failure; PAD, peripheral artery disease.

In Cox regression analysis, experiencing a cardiovascular event, regardless of type, was found to be associated with elevated all-cause mortality. However, the initial occurrence of heart failure (HF) was linked to a greater risk, independently of age and sex, compared to coronary heart disease (CHD), peripheral artery disease (PAD), or cerebrovascular disease (**Figure 4**).

## 4 Discussion

In this longitudinal retrospective analysis of a large cohort of individuals with T2D diabetes in primary prevention from a primary care setting in a western Mediterranean region, we found that HF and PAD substantially contribute to CVD. We also observed a considerable influence of sex and age on overall cardiovascular risk and the form of the initial clinical presentation of CVD. Within our cohort, we observed that men exhibited a higher susceptibility to overall CVD, CHD, cerebrovascular disease, and PAD compared to women. However, men demonstrated a lower risk for HF across all age groups. CHD emerged as the predominant CVD presentation among men and younger individuals, whereas HF predominated among women and older participants.

We found an annual incidence rate of CVD of around 4%. This rate was similar to that reported by Shah et al. in a recent study that included 34,198 individuals with T2D free of cardiovascular disease from primary care settings in England but higher than the 1 to 2% per year observed in other studies (1, 15–20). Several factors could explain the variability between studies; however, one of the most relevant might be the number and type of outcomes included in the CVD definition. As in the study mentioned above by Shah et al. (1), we applied a broad definition that included HF and PAD. Notably, both conditions combined accounted for more than 50% of the first-ever individual events in our cohort and 1 in 3 in the study of Shah et al. (1). In contrast, in most previous works, CVD was restricted to coronary and cerebrovascular events. Only a few studies included HF (17), and even fewer included PAD (17, 19).

Our findings that HF and PAD are leading contributors to T2D cardiovascular burden reinforce the importance of considering them in routine clinical practice and risk estimation strategies.

That being said, there were considerable variations in the absolute risk for the various manifestations of CVD by sex and age.

First, as in previous studies, we observed that women had lower overall CVD risk and lower risk for atherosclerotic diseases than men, independent of age (6). In contrast, we found higher incidence rates for HF in women than men at any age. Previous studies assessing sex differences in absolute HF risk in individuals with T2D are conflicting. In older studies, a higher absolute risk was observed in men (21, 22), whereas, in more contemporary works, women showed numerically higher rates for HF than men (12, 21–23). Nonetheless, none of these studies focused primarily on first events or considered the full effect of age. Thus, our results further reinforce the importance of HF as a primary driver of CVD in women with T2D. Mechanisms explaining a higher HF risk in women are not yet fully elucidated and might include differences in cardiac structure, function, and metabolism, differential myocardial response to classical cardiovascular risk factors, and exposure in women to unique risks, such as pre-eclampsia (24–26). It should also underscored that, in our cohort, the presentation of cardiovascular disease as HF was associated with a higher risk for all-cause mortality than other CVD subtypes, independently of age and sex. These results, which are in line with previous literature suggesting a profound impact of HF on overall survival, might further reinforce the importance of strategies primarily addressing its prevention (27).

Second, although sex patterns for CVD’s first manifestation were consistent across age groups, atherosclerotic diseases, mainly CHD, accounted for most premature events in both men and women. In contrast, HF predominated in the aging population of both sexes. This finding agrees with previous epidemiological studies conducted in the general population, showing that HF is disproportionally distributed among older adults, with a prevalence that doubles for each decade of life (28).

Finally, we observed a sex-differential association between hypertension, obesity, socio-economic status, and incident cardiovascular disease. In our work, as in previous studies, the relative contribution of these factors was higher for women than for men (29, 30). Similarly, as in earlier cohort studies, in our work, the relative risk associated with classical cardiovascular risk factors, metabolic control, and end-organ damage was higher in younger vs. older individuals (31). These data have important clinical implications. First, it should help evaluate and discuss cardiovascular risks and preventive strategies in the clinical setting and facilitate shared decision-making from a more individualised perspective. Second, the evidence of large sex and age differences in CVD epidemiology and the possibility of a sex and age-differential impact of classical and non-classical cardiovascular risk factors among individuals with T2D should prompt us to evaluate the ongoing cardiovascular preventive strategies. Of note, current cardiovascular risk management in T2D is based on evidence from clinical trials mainly focused on coronary and cerebrovascular disease prevention conducted in predominantly middle-aged populations where women were frequently underrepresented (32–36). Although this approach has been proven to be highly effective in diminishing cardiovascular morbidity and mortality, T2D-related cardiovascular burdens might be further reduced by considering sex and age-specific risks and by modulating medical interventions in accordance.

In this regard, hypertension treatment dramatically reduces the incidence of HF and stroke but has a smaller impact on CHD (37, 38). Furthermore, observational data suggested that cardiovascular risk in women rises at lower blood pressure thresholds and that hypertension might confer a greater risk for HF in women than in men (30). In this same line, the recent Systolic Blood Pressure Intervention Trial (SPRINT), which unfortunately excluded participants with T2D, suggested a more significant benefit of lowering blood pressure goals in older vs. younger participants (37). On the other hand, lowering LDL-cholesterol dose-dependently decreases the incidence of atherosclerotic events, especially CHD (39). However, statin treatment is associated with limited improvements in preventing first non-fatal HF hospitalisations (40). Finally, the relative benefits of the newest T2D therapies vary. While sodium-glucose transport type 2 inhibitors have been shown to significantly impact HF, the cardiovascular benefits of glucagon-like peptide 1 agonists mostly rely on preventing atherosclerotic diseases (41). Thus, it could be speculated that the absolute cardiovascular benefits of these newer therapies might also vary by age and sex. Unfortunately, no clinical trials have been designed to primarily explore sex or age-specific targets or therapies for CVD prevention. Our findings further reinforce the need to fill this lack of evidence on these clinically relevant issues.

Our study has several limitations. First, diagnoses were recorded by treating physicians based on ICD-9/ICD-10 codes. Thus, some inter-individual variability in definitions cannot be excluded. Also, over 23% of Catalonians receive health care outside the National Health Care System (*Institut Català de la Salut*) due to health agreements between the government and the private sector. This population is not included in the SIDIAP database. Nonetheless, the data quality of the SIDIAP database has been previously validated, and more specifically, data on CVD has proven to be of high quality and suitable for epidemiological studies (42, 43). Second, there was a substantial proportion of missing values for some variables (including BMI, blood pressure, or lipid profile). Therefore, the analysis assessing the association between risk factors and cardiovascular disease should be interpreted cautiously. Thirdly, it’s important to note that in Spain, sodium-glucose transporters 2 inhibitors and long-acting glucagon-like 1 peptide analogs were not accessible at the baseline study date, and our dataset does not extend beyond 2016. Therefore, we cannot assess whether these newer agents’ prescriptions might impact cardiovascular disease incidence and presentation. However, it is worth highlighting that the utilization of these advanced therapies remains minimal within the Catalonian population. As of 2018, their usage had stayed within 5% (41). Further studies comparing cardiovascular disease and its main subtypes incidence before and after full implementation of SGLT2-i and GLP1-analogs in the clinical practice help to delineate their impact in a real-world setting. Finally, the specific cause of death was only available for a limited proportion of study participants.

## 5 Conclusion

In conclusion, our study demonstrated that HF and PAD are significant contributors to cardiovascular disease burden in subjects with T2D and that, as in the general population, in women and older individuals, CVD more frequently manifests as HF. This epidemiological evidence should be considered in clinical practice and risk estimation strategies and might be relevant in designing future clinical trials.

## Data availability statement

The original contributions presented in the study are included in the article/**Supplementary Material**. Further inquiries can be directed to the corresponding authors.

## Ethics statement

The Ethics Committee of the Primary Healthcare University Research Institute (IDIAP), Jordi Gol (Barcelona, Spain), approved the study (code P17/087). The study was conducted in accordance with the local legislation and institutional requirements. Minimal risk research permitted the authors to conduct the investigation without obtaining informed consent from patients. Data was anonymized and is untraceable.

## Author contributions

AJ: Writing – original draft. BV: Data curation, Methodology, Writing – review & editing. MM: Conceptualization, Investigation, Writing – review & editing. JR: Formal analysis, Methodology, Writing – review & editing. DM: Conceptualization, Funding acquisition, Investigation, Writing – review & editing. JF: Conceptualization, Funding acquisition, Investigation, Writing – review & editing. EO: Conceptualization, Funding acquisition, Investigation, Writing – review & editing.
