# Supplementary material for: Sex and age significantly modulate cardiovascular disease presentation in type 2 diabetes: a large population-based cohort study
Source: Front Endocrinol (Lausanne). 2024 May 17;15:1344007. doi: 10.3389/fendo.2024.1344007 (PMC11140096; doi:10.3389/fendo.2024.1344007)
Supplement: Supplementary file 1 [file DataSheet_1.docx]

**Supplementary Table 1: International Classification of Diseases and Related Health Problems codes for retinopathy, nephropathy, polyneuropathy, and hypertension, used in this report.**

| **ICD9/10** | **CODE** | **Description (native Catalan language)** | **Comorbiditie or complication** |
| --- | --- | --- | --- |
| CIM9 | 401 | Hipertensió essencial | Hypertension |
| CIM9 | 4010 | Hipertensió essencial maligna | Hypertension |
| CIM9 | 4011 | Hipertensió essencial benigna | Hypertension |
| CIM9 | 4019 | Hipertensió essencial no especificada | Hypertension |
| CIM9 | 402 | Cardiopatia hipertensiva | Hypertension |
| CIM9 | 4020 | Cardiopatia hipertensiva maligna | Hypertension |
| CIM9 | 40200 | Cardiopatia hipertensiva maligna sense insuficiència cardíaca | Hypertension |
| CIM9 | 40201 | Cardiopatia hipertensiva maligna amb insuficiència cardíaca | Hypertension |
| CIM9 | 4021 | Cardiopatia hipertensiva benigna | Hypertension |
| CIM9 | 40210 | Cardiopatia hipertensiva benigna sense insuficiència cardíaca | Hypertension |
| CIM9 | 40211 | Cardiopatia hipertensiva benigna amb insuficiència cardíaca | Hypertension |
| CIM9 | 4029 | Cardiopatia hipertensiva no especificada | Hypertension |
| CIM9 | 40290 | Cardiopatia hipertensiva no especificada sense insuficiència cardíaca | Hypertension |
| CIM9 | 40291 | Cardiopatia hipertensiva no especificada amb insuficiència cardíaca | Hypertension |
| CIM9 | 403 | Malaltia renal crònica hipertensiva | Hypertension |
| CIM9 | 4030 | Malaltia renal crònica hipertensiva maligna | Hypertension |
| CIM9 | 40300 | Malaltia renal crònica hipertensiva maligna amb malaltia renal crònica d'estadi I a estadi IV o estadi NE | Hypertension |
| CIM9 | 40301 | Malaltia renal crònica hipertensiva maligna amb malaltia renal crònica d'estadi V o malaltia renal terminal | Hypertension |
| CIM9 | 4031 | Malaltia renal crònica hipertensiva benigna | Hypertension |
| CIM9 | 40310 | Malaltia renal crònica hipertensiva benigna amb malaltia renal crònica d'estadi I a estadi IV o estadi NE | Hypertension |
| CIM9 | 40311 | Malaltia renal crònica hipertensiva benigna amb malaltia renal crònica d'estadi V o malaltia renal terminal | Hypertension |
| CIM9 | 4039 | Malaltia renal crònica hipertensiva no especificada | Hypertension |
| CIM9 | 40390 | Malaltia renal crònica hipertensiva no especificada amb malaltia renal crònica d'estadi I a estadi IV o estadi NE | Hypertension |
| CIM9 | 40391 | Malaltia renal crònica hipertensiva no especificada amb malaltia renal crònica d'estadi V o malaltia renal terminal | Hypertension |
| CIM9 | 404 | Cardiopatia hipertensiva i malaltia renal crònica hipertensiva | Hypertension |
| CIM9 | 4040 | Cardiopatia hipertensiva i malaltia renal crònica hipertensiva malignes | Hypertension |
| CIM9 | 40400 | Cardiopatia hipertensiva i malaltia renal crònica hipertensiva malignes sense insuficiència cardíaca i amb malaltia renal crònica d'estadi I a estadi IV o estadi NE | Hypertension |
| CIM9 | 40401 | Cardiopatia hipertensiva i malaltia renal crònica hipertensiva malignes amb insuficiència cardíaca i amb malaltia renal crònica d'estadi I a estadi IV o estadi NE | Hypertension |
| CIM9 | 40402 | Cardiopatia hipertensiva i malaltia renal crònica hipertensiva malignes sense insuficiència cardíaca i amb malaltia renal crònica d'estadi V o malaltia renal terminal | Hypertension |
| CIM9 | 40403 | Cardiopatia hipertensiva i malaltia renal crònica hipertensiva malignes amb insuficiència cardíaca i malaltia renal crònica d'estadi V o malaltia renal terminal | Hypertension |
| CIM9 | 4041 | Cardiopatia hipertensiva i malaltia renal crònica hipertensiva benignes | Hypertension |
| CIM9 | 40410 | Cardiopatia hipertensiva i malaltia renal crònica hipertensiva benignes sense insuficiència cardíaca i amb malaltia renal crònica d'estadi I a estadi IV o estadi NE | Hypertension |
| CIM9 | 40411 | Cardiopatia hipertensiva i malaltia renal crònica hipertensiva benignes amb insuficiència cardíaca i amb malaltia renal crònica d'estadi I a estadi IV o estadi NE | Hypertension |
| CIM9 | 40412 | Cardiopatia hipertensiva i malaltia renal crònica hipertensiva benignes sense insuficiència cardíaca i amb malaltia renal crònica d'estadi V o malaltia renal terminal | Hypertension |
| CIM9 | 40413 | Cardiopatia hipertensiva i malaltia renal crònica hipertensiva benignes amb insuficiència cardíaca i malaltia renal crònica d'estadi V o malaltia renal terminal | Hypertension |
| CIM9 | 4049 | Cardiopatia hipertensiva i malaltia renal crònica hipertensiva no especificades | Hypertension |
| CIM9 | 40490 | Cardiopatia hipertensiva i malaltia renal crònica hipertensiva no especificades sense insuficiència cardíaca i amb malaltia renal crònica d'estadi I a estadi IV o estadi NE | Hypertension |
| CIM9 | 40491 | Cardiopatia hipertensiva i malaltia renal crònica hipertensiva no especificades amb insuficiència cardíaca i amb malaltia renal crònica d'estadi I a estadi IV o estadi NE | Hypertension |
| CIM9 | 40492 | Cardiopatia hipertensiva i malaltia renal crònica hipertensiva no especificades sense insuficiència cardíaca i amb malaltia renal crònica d'estadi V o malaltia renal terminal | Hypertension |
| CIM9 | 40493 | Cardiopatia hipertensiva i malaltia renal crònica hipertensiva no especificades amb insuficiència cardíaca i malaltia renal crònica d'estadi V o malaltia renal terminal | Hypertension |
| CIM9 | 405 | Hipertensió secundària | Hypertension |
| CIM9 | 4050 | Hipertensió secundària maligna | Hypertension |
| CIM9 | 40501 | Hipertensió secundària maligna renovascular | Hypertension |
| CIM9 | 40509 | Altres tipus d'hipertensió secundària maligna | Hypertension |
| CIM9 | 4051 | Hipertensió secundària benigna | Hypertension |
| CIM9 | 40511 | Hipertensió secundària benigna renovascular | Hypertension |
| CIM9 | 40519 | Altres tipus d'hipertensió secundària benigna | Hypertension |
| CIM9 | 4059 | Hipertensió secundària no especificada | Hypertension |
| CIM9 | 40591 | Hipertensió secundària no especificada renovascular | Hypertension |
| CIM9 | 40599 | Altres tipus d'hipertensió secundària no especificada | Hypertension |
| CIM10 | I10 | HIPERTENSIÓ ESSENCIAL (PRIMÀRIA) | Hypertension |
| CIM10 | I11 | HIPERTENSIÓ | Hypertension |
| CIM10 | I11.0 | HIPERTENSIÓ | Hypertension |
| CIM10 | I11.9 | HIPERTENSIÓ | Hypertension |
| CIM10 | I12 | HIPERTENSIÓ | Hypertension |
| CIM10 | I12.0 | HIPERTENSIÓ | Hypertension |
| CIM10 | I12.9 | HIPERTENSIÓ | Hypertension |
| CIM10 | I13 | HIPERTENSIÓ | Hypertension |
| CIM10 | I13.0 | HIPERTENSIÓ | Hypertension |
| CIM10 | I13.1 | HIPERTENSIÓ | Hypertension |
| CIM10 | I13.2 | HIPERTENSIÓ | Hypertension |
| CIM10 | I13.9 | HIPERTENSIÓ | Hypertension |
| CIM10 | I15 | HIPERTENSIÓ SECUNDÀRIA | Hypertension |
| CIM10 | I15.0 | HIPERTENSIÓ RENOVASCULAR | Hypertension |
| CIM10 | I15.1 | HIPERTENSIÓ SECUNDÀRIA A ALTRES TRASTORNS RENALS | Hypertension |
| CIM10 | I15.2 | HIPERTENSIÓ SECUNDÀRIA A TRASTORNS ENDOCRINS | Hypertension |
| CIM10 | I15.8 | ALTRES TIPUS D'HIPERTENSIÓ SECUNDÀRIA | Hypertension |
| CIM10 | I15.9 | HIPERTENSIÓ SECUNDÀRIA, NO ESPECIFICADA | Hypertension |
| CIM9 | 3620 | Retinopatia diabètica | Diabetic Retinopathy |
| CIM9 | 36201 | Retinopatia diabètica de fons | Diabetic Retinopathy |
| CIM9 | 36202 | Retinopatia diabètica proliferativa | Diabetic Retinopathy |
| CIM9 | 36203 | Retinopatia diabètica no proliferativa no especificada | Diabetic Retinopathy |
| CIM9 | 36204 | Retinopatia diabètica no proliferativa lleu | Diabetic Retinopathy |
| CIM9 | 36205 | Retinopatia diabètica no proliferativa moderada | Diabetic Retinopathy |
| CIM9 | 36206 | Retinopatia diabètica no proliferativa greu | Diabetic Retinopathy |
| CIM9 | 36207 | Edema macular diabètic | Diabetic Retinopathy |
| CIM9 | 36271 | Distròfia retinal en lipidosis sistèmiques o cerebroretinals | Diabetic Retinopathy |
| CIM9 | 36641 | Cataracta diabètica | Diabetic Retinopathy |
| CIM10 | H36.0 | RETINOPATIA DIABÈTICA (E10-E14+ AMB QUART CARÀCTER COMÚ .3) | Diabetic Retinopathy |
| CIM9 | 25060 | Diabetis amb manifestacions neurològiques, tipus II o tipus no especificat, no esmentada com a incontrolada | Diabetic Neuropathy |
| CIM9 | 3371 | Neuropatia autonòmica perifèrica en trastorns classificats en un altre lloc | Diabetic Neuropathy |
| CIM9 | 3540 | Síndrome del túnel carpià | Diabetic Neuropathy |
| CIM9 | 3541 | Altres lesions del nervi medià | Diabetic Neuropathy |
| CIM9 | 3542 | Lesió del nervi cubital | Diabetic Neuropathy |
| CIM9 | 3543 | Lesió del nervi radial | Diabetic Neuropathy |
| CIM9 | 3544 | Causàlgia de l'extremitat superior | Diabetic Neuropathy |
| CIM9 | 3545 | Mononeuritis múltiple | Diabetic Neuropathy |
| CIM9 | 3548 | Altres mononeuritis del membre superior | Diabetic Neuropathy |
| CIM9 | 3549 | Mononeuritis del membre superior no especificada | Diabetic Neuropathy |
| CIM9 | 355 | Mononeuritis de l'extremitat inferior de localització no especificada | Diabetic Neuropathy |
| CIM9 | 3550 | Lesió del nervi ciàtic | Diabetic Neuropathy |
| CIM9 | 3551 | Meràlgia parestèsica | Diabetic Neuropathy |
| CIM9 | 3552 | Altres lesions del nervi femoral | Diabetic Neuropathy |
| CIM9 | 3553 | Lesió del nervi popliti extern | Diabetic Neuropathy |
| CIM9 | 3554 | Lesió del nervi popliti intern | Diabetic Neuropathy |
| CIM9 | 3555 | Síndrome del túnel tarsià | Diabetic Neuropathy |
| CIM9 | 3556 | Lesió del nervi plantar | Diabetic Neuropathy |
| CIM9 | 3557 | Altres mononeuritis de l'extremitat inferior | Diabetic Neuropathy |
| CIM9 | 35571 | Causàlgia de l'extremitat inferior | Diabetic Neuropathy |
| CIM9 | 35579 | Altres mononeuritis del membre inferior | Diabetic Neuropathy |
| CIM9 | 3558 | Mononeuritis del membre inferior no especificada | Diabetic Neuropathy |
| CIM9 | 3559 | Mononeuritis de localització no especificada | Diabetic Neuropathy |
| CIM9 | 3572 | Polineuropatia diabètica | Diabetic Neuropathy |
| CIM10 | E11.4 | DIABETIS MELLITUS NO INSULINODEPENDENT, AMB COMPLICACIONS NEUROLÒGIQUES | Diabetic Neuropathy |
| CIM10 | G63.2 | POLINEUROPATIA DIABÈTICA (E10-E14+ AMB QUART CARÀCTER COMÚ .4) | Diabetic Neuropathy |
| CIM10 | G99.0 | NEUROPATIA AUTONÒMICA EN MALALTIES METABÒLIQUES I ENDOCRINES | Diabetic Neuropathy |
| CIM9 | 25040 | Diabetis amb manifestacions renals, tipus II o tipus no especificat, no esmentada com a incontrolada | Diabetic Nephropathy |
| CIM9 | 58381 | Nefritis i nefropatia, no especificades com a agudes o cròniques, en malalties classificades en un altre lloc | Diabetic Nephropathy |
| CIM9 | 585 | Malaltia renal crònica | Diabetic Nephropathy |
| CIM9 | 5851 | Malaltia renal crònica, estadi I | Diabetic Nephropathy |
| CIM9 | 5852 | Malaltia renal crònica, estadi II (lleu) | Diabetic Nephropathy |
| CIM9 | 5853 | Malaltia renal crònica, estadi III (moderada) | Diabetic Nephropathy |
| CIM9 | 5854 | Malaltia renal crònica, estadi IV (greu) | Diabetic Nephropathy |
| CIM9 | 5855 | Malaltia renal crònica, estadi V | Diabetic Nephropathy |
| CIM9 | 5856 | Malaltia renal terminal | Diabetic Nephropathy |
| CIM9 | 5859 | Malaltia renal crònica no especificada | Diabetic Nephropathy |
| CIM9 | 7910 | Proteïnúria | Diabetic Nephropathy |
| CIM10 | E11.2 | DIABETIS MELLITUS NO INSULINODEPENDENT, AMB COMPLICACIONS RENALS | Diabetic Nephropathy |
| CIM10 | N08.3 | TRANSTORNS GLOMERULARS DIABETIS MELLITUS | Diabetic Nephropathy |
| CIM10 | N18 | INSUFICIÈNCIA RENAL CRÒNICA | Diabetic Nephropathy |
| CIM10 | N18.0 | INSUFICIÈNCIA RENAL TERMINAL | Diabetic Nephropathy |
| CIM10 | N18.8 | ALTRES INSUFICIÈNCIES RENALS CRÒNIQUES | Diabetic Nephropathy |
| CIM10 | N18.9 | INSUFICIÈNCIA RENAL CRÒNICA, NO ESPECIFICADA | Diabetic Nephropathy |
| CIM10 | R80 | PROTEÏNURIA AÏLLADA | Diabetic Nephropathy |
| CIM9 | V420 | Ronyó substituït per trasplantament | Diabetic Nephropathy |
| CIM9 | V560 | Assistència per a diàlisi extracorpòria | Diabetic Nephropathy |
| CIM10 | Z49 | ATENCIONS RELATIVES AL PROCEDIMENT DE DIÀLISI | Diabetic Nephropathy |
| CIM10 | Z49.0 | PREPARACIÓ PER A LA DIÀLISI | Diabetic Nephropathy |
| CIM10 | Z49.1 | DIÀLISIS EXTRACORPÒRIA | Diabetic Nephropathy |
| CIM10 | Z49.2 | ALTRES DIÀLISIS | Diabetic Nephropathy |
| CIM10 | Z94.0 | TRASPLANTAMENT DE RONYÓ | Diabetic Nephropathy |

**Supplementary Table 2**: **International Classification of Diseases and Related Health Problems codes for cardiovascular disease used in this report**

| **ICD9/10** | **CODE** | **Description (native Catalan language)** | **TERRITORY** | **MANIFESTATION** |
| --- | --- | --- | --- | --- |
| CIM10 | G45 | ATACS D’ISQUÈMIA CEREBRAL TRANSITÒRIA I SÍNDROMES AFINS | CEREBROVASCULAR | TRANSIENT ISCHEMIC ATTACK (TIA) |
| CIM10 | G45.0 | SÍNDROME ARTERIAL VERTEBROBASILAR | CEREBROVASCULAR | TRANSIENT ISCHEMIC ATTACK (TIA) |
| CIM10 | G45.1 | SÍNDROME DE L’ARTÈRIA CARÒTIDA (HEMISFÈRIC) | CEREBROVASCULAR | TRANSIENT ISCHEMIC ATTACK (TIA) |
| CIM10 | G45.2 | SÍNDROMES ARTERIALS PRECEREBRALS BILATERALS I MÚLTIPLES | CEREBROVASCULAR | TRANSIENT ISCHEMIC ATTACK (TIA) |
| CIM10 | G45.3 | AMAUROSI FUGAÇ | CEREBROVASCULAR | TRANSIENT ISCHEMIC ATTACK (TIA) |
| CIM10 | G45.4 | AMNÈSIA GLOBAL TRANSITÒRIA | CEREBROVASCULAR | TRANSIENT ISCHEMIC ATTACK (TIA) |
| CIM10 | G45.8 | ALTRES ISQUÈMIES CEREBRALS TRANSITÒRIES I SÍNDROMES AFINS | CEREBROVASCULAR | TRANSIENT ISCHEMIC ATTACK (TIA) |
| CIM10 | G45.9 | ISQUÈMIA CEREBRAL TRANSITÒRIA, SENSE ALTRA ESPECIFICACIÓ | CEREBROVASCULAR | TRANSIENT ISCHEMIC ATTACK (TIA) |
| CIM10 | G46 | SÍNDROMES VASCULARS ENCEFÀLIQUES EN MALALTIES CEREBROVASCULARS (I60- 167+) | CEREBROVASCULAR | ISCHEMIC STROKE |
| CIM10 | G46.0 | SÍNDROME DE L’ARTÈRIA CEREBRAL MITJANA (I66.0+) | CEREBROVASCULAR | ISCHEMIC STROKE |
| CIM10 | G46.1 | SÍNDROME DE L’ARTÈRIA CEREBRAL ANTERIOR (I66.1+) | CEREBROVASCULAR | ISCHEMIC STROKE |
| CIM10 | G46.2 | SÍNDROME DE L’ARTÈRIA CEREBRAL POSTERIOR (I66.2+) | CEREBROVASCULAR | ISCHEMIC STROKE |
| CIM10 | G46.3 | SÍNDROMES APOPLÈTIQUES DE LA TIJA ENCEFÀLICA (I60-I67+) | CEREBROVASCULAR | ISCHEMIC STROKE |
| CIM10 | G46.4 | SÍNDROME D’INFART CEREBEL·LÓS (I60-I67+) | CEREBROVASCULAR | ISCHEMIC STROKE |
| CIM10 | G46.5 | SÍNDROME LACUNAR MOTOR PURA (I60-I67+) | CEREBROVASCULAR | ISCHEMIC STROKE |
| CIM10 | G46.6 | SÍNDROME LACUNAR SENSORIAL PURA (I60-I67+) | CEREBROVASCULAR | ISCHEMIC STROKE |
| CIM10 | G46.7 | ALTRES SÍNDROMES LACUNARS (I60-I67+) | CEREBROVASCULAR | ISCHEMIC STROKE |
| CIM10 | G46.8 | ALTRES SÍNDROMES VASCULARS ENCEFÀLICS EN MALALTIES CEREBROVASCULARS (I160-167+) | CEREBROVASCULAR | ISCHEMIC STROKE |
| CIM10 | I11.0 | MALALTIA CARDÍACA HIPERTENSIVA AMB INSUFICIÈNCIA CARDÍACA (CONGESTIVA) | HEART FAILURE | HEART FAILURE_CONGESTIVE |
| CIM10 | I13.0 | MALALTIA CARDIORENAL HIPERTENSIVA AMB INSUFICIÈNCIA CARDÍACA (CONGESTIVA) | HEART FAILURE | HEART FAILURE_CONGESTIVE |
| CIM10 | I13.2 | MALALTIA CARDIORENAL HIPERTENSIVA AMB INSUFICIÈNCIA CARDÍACA (CONGESTIVA) I INSUFICIÈNCIA RENAL | HEART FAILURE | HEART FAILURE_CONGESTIVE |
| CIM10 | I20 | ANGINA DE PIT | CORONARY | ANGOR |
| CIM10 | I20.0 | ANGINA INESTABLE | CORONARY | ANGOR |
| CIM10 | I20.1 | ANGINA DE PIT AMB ESPASME DOCUMENTAT | CORONARY | ANGOR |
| CIM10 | I20.8 | ALTRES FORMES ESPECÍFIQUES D’ANGINA DE PIT | CORONARY | ANGOR |
| CIM10 | I20.9 | ANGINA DE PIT, NO ESPECIFICADA | CORONARY | ANGOR |
| CIM10 | I21 | INFART AGUT DE MIOCARDI | CORONARY | MIOCARDIAL INFARCTION |
| CIM10 | I21.0 | INFART TRANSMURAL AGUT DE MIOCARDI DE LA PARET ANTERIOR | CORONARY | MIOCARDIAL INFARCTION |
| CIM10 | I21.1 | INFART TRANSMURAL AGUT DE MIOCARDI DE LA PARET INFERIOR | CORONARY | MIOCARDIAL INFARCTION |
| CIM10 | I21.2 | INFART TRANSMURAL AGUT DE MIOCARDI D’ALTRES LLOCS | CORONARY | MIOCARDIAL INFARCTION |
| CIM10 | I21.3 | INFART TRANSMURAL AGUT DE MIOCARDI, DE LOCALITZACIÓ NO ESPECIFICADA | CORONARY | MIOCARDIAL INFARCTION |
| CIM10 | I21.4 | INFART SUBENDOCÀRDIC AGUT DE MIOCARDI | CORONARY | MIOCARDIAL INFARCTION |
| CIM10 | I21.9 | INFART AGUT DE MIOCARDI, SENSE ALTRA ESPECIFICACIÓ | CORONARY | MIOCARDIAL INFARCTION |
| CIM10 | I22 | INFART SUBSEGÜENT DEL MIOCARDI | CORONARY | MIOCARDIAL INFARCTION |
| CIM10 | I22.0 | INFART SUBSEGÜENT DE MIOCARDI DE LA PARET ANTERIOR | CORONARY | MIOCARDIAL INFARCTION |
| CIM10 | I22.1 | INFART SUBSEGÜENT DE MIOCARDI DE LA PARET INFERIOR | CORONARY | MIOCARDIAL INFARCTION |
| CIM10 | I22.8 | INFART SUBSEGÜENT DE MIOCARDI D’ALTRES LLOCS | CORONARY | MIOCARDIAL INFARCTION |
| CIM10 | I22.9 | INFART SUBSEGÜENT DE MIOCARDI, DE PART NO ESPECIFICADA | CORONARY | MIOCARDIAL INFARCTION |
| CIM10 | I23 | ALGUNES COMPLICACIONS POSTERIORS A L’INFART AGUT DE MIOCARDI | CORONARY | MIOCARDIAL INFARCTION |
| CIM10 | I23.0 | HEMOPERICARDI COM A COMPLICACIÓ PRESENT I POSTERIOR A L’INFART AGUT DE MIOCARDI | CORONARY | MIOCARDIAL INFARCTION |
| CIM10 | I23.1 | DEFECTE DE L’ENVÀ AURICULAR COM A COMPLICACIÓ PRESENT I POSTERIOR A L’INFART DE MIOCARDI | CORONARY | MIOCARDIAL INFARCTION |
| CIM10 | I23.2 | DEFECTE DE L’ENVÀ VENTRICULAR COM A COMPLICACIÓ PRESENT I POSTERIOR A L’INFART DE MIOCARDI | CORONARY | MIOCARDIAL INFARCTION |
| CIM10 | I23.3 | RUPTURA DE LA PARET CARDÍACA SENSE HEMOPERICARDI, COM A COMPLICACIÓ PRESENT I POSTERIOR A L’INFART AGUT DE MIOCARDI | CORONARY | MIOCARDIAL INFARCTION |
| CIM10 | I23.4 | RUPTURA DE CORDES TENDINOSES COM A COMPLICACIÓ PRESENT I POSTERIOR A L’INFART AGUT DE MIOCARDI | CORONARY | MIOCARDIAL INFARCTION |
| CIM10 | I23.5 | RUPTURA DE MÚSCUL PAPIL·LAR COM A COMPLICACIÓ PRESENT I POSTERIOR A L’INFART AGUT DE MIOCARDI | CORONARY | MIOCARDIAL INFARCTION |
| CIM10 | I23.6 | TROMBOSI AURICULAR (APÈNDIX AURICULAR) I VENTRICULAR COM A COMPLICACIÓ PRESENT I POSTERIOR A L’INFART AGUT DE MIOCARDI | CORONARY | MIOCARDIAL INFARCTION |
| CIM10 | I23.8 | ALTRES COMPLICACIONS POSTERIORS A L’INFART AGUT DE MIOCARDI | CORONARY | MIOCARDIAL INFARCTION |
| CIM10 | I24 | ALTRES MALALTIES ISQUÈMIQUES AGUDES DEL COR | CORONARY | ANGOR |
| CIM10 | I24.0 | TROMBOSI CORONÀRIA QUE NO PROVOCA UN INFART DE MIOCARDI | CORONARY | INDETERMINATE CORONARY ARTERY DISEASE |
| CIM10 | I24.8 | ALTRES FORMES DE MALALTIA ISQUÈMICA AGUDA DEL COR | CORONARY | INDETERMINATE CORONARY ARTERY DISEASE |
| CIM10 | I24.9 | MALALTIA ISQUÈMICA AGUDA DEL COR, NO ESPECIFICADA | CORONARY | ANGOR |
| CIM10 | I25 | MALALTIA ISQUÈMICA CRÒNICA DEL COR | CORONARY | INDETERMINATE CORONARY ARTERY DISEASE |
| CIM10 | I25.0 | MALALTIA CARDIOVASCULAR ATEROSCLERÒTICA, DESCRITA D’AQUESTA MANERA | CORONARY | INDETERMINATE CORONARY ARTERY DISEASE |
| CIM10 | I25.1 | MALALTIA ATEROSCLERÒTICA DEL COR | CORONARY | INDETERMINATE CORONARY ARTERY DISEASE |
| CIM10 | I25.2 | INFART ANTIC DE MIOCARDI | CORONARY | MIOCARDIAL INFARCTION |
| CIM10 | I25.5 | MIOCARDIOPATIA ISQUÈMICA | CORONARY | INDETERMINATE CORONARY ARTERY DISEASE |
| CIM10 | I25.6 | ISQUÈMIA SILENT DE MIOCARDI | CORONARY | INDETERMINATE CORONARY ARTERY DISEASE |
| CIM10 | I25.8 | ALTRES FORMES DE MALALTIA ISQUÈMICA CRÒNICA DEL COR | CORONARY | INDETERMINATE CORONARY ARTERY DISEASE |
| CIM10 | I25.9 | MALALTIA ISQUÈMICA CRÒNICA DEL COR, NO ESPECIFICADA | CORONARY | INDETERMINATE CORONARY ARTERY DISEASE |
| CIM10 | I50.0 | INSUFICIÈNCIA CARDÍACA CONGESTIVA | HEART FAILURE | HEART FAILURE_CONGESTIVE |
| CIM9 | 4280 | Insuficiència cardíaca congestiva no especificada | HEART FAILURE | HEART FAILURE_CONGESTIVE |
| CIM9 | 42821 | Insuficiència cardíaca sistòlica aguda | HEART FAILURE | HEART FAILURE_CONGESTIVE |
| CIM9 | 42831 | Insuficiència cardíaca diastòlica aguda | HEART FAILURE | HEART FAILURE_CONGESTIVE |
| CIM9 | 42841 | Insuficiència cardíaca combinada sistòlica/diastòlica aguda | HEART FAILURE | HEART FAILURE_CONGESTIVE |
| CIM10 | I50 | INSUFICIÈNCIA CARDÍACA | HEART FAILURE | HEART FAILURE_OTHERS |
| CIM10 | I61 | HEMORRÀGIA INTRAENCEFÀLICA | CEREBROVASCULAR | HEMORRHAGIC STROKE |
| CIM10 | I61.0 | HEMORRÀGIA INTRACEREBRAL DE L’HEMISFERI SUBCORTICAL | CEREBROVASCULAR | HEMORRHAGIC STROKE |
| CIM10 | I61.1 | HEMORRÀGIA INTRACEREBRAL DE L’HEMISFERI CORTICAL | CEREBROVASCULAR | HEMORRHAGIC STROKE |
| CIM10 | I61.2 | HEMORRÀGIA INTRACEREBRAL DE L’HEMISFERI, NO ESPECIFICADA | CEREBROVASCULAR | HEMORRHAGIC STROKE |
| CIM10 | I61.3 | HEMORRÀGIA INTRAENCEFÀLICA EN LA TIJA CEREBRAL | CEREBROVASCULAR | HEMORRHAGIC STROKE |
| CIM10 | I61.4 | HEMORRÀGIA INTRAENCEFÀLICA EN EL CEREBEL | CEREBROVASCULAR | HEMORRHAGIC STROKE |
| CIM10 | I61.5 | HEMORRÀGIA INTRAENCEFÀLICA, INTRAVENTRICULAR | CEREBROVASCULAR | HEMORRHAGIC STROKE |
| CIM10 | I61.6 | HEMORRÀGIA INTRAENCEFÀLICA DE LOCALITZACIONS MÚLTIPLES | CEREBROVASCULAR | HEMORRHAGIC STROKE |
| CIM10 | I61.8 | ALTRES HEMORRÀGIES INTRAENCEFÀLIQUES | CEREBROVASCULAR | HEMORRHAGIC STROKE |
| CIM10 | I61.9 | HEMORRÀGIA INTRAENCEFÀLICA, NO ESPECIFICADA | CEREBROVASCULAR | HEMORRHAGIC STROKE |
| CIM10 | I63 | INFART CEREBRAL | CEREBROVASCULAR | ISCHEMIC STROKE |
| CIM10 | I63.0 | INFART CEREBRAL SECUNDARI A TROMBOSI D’ARTÈRIES PRECEREBRALS | CEREBROVASCULAR | ISCHEMIC STROKE |
| CIM10 | I63.1 | INFART CEREBRAL SECUNDARI A EMBÒLIA D’ARTÈRIES PRECEREBRALS | CEREBROVASCULAR | ISCHEMIC STROKE |
| CIM10 | I63.2 | INFART CEREBRAL SECUNDARI A OCLUSIÓ O ESTENOSI NO ESPECIFICADA D’ARTÈRIES PRECEREBRALS | CEREBROVASCULAR | ISCHEMIC STROKE |
| CIM10 | I63.3 | INFART CEREBRAL SECUNDARI A TROMBOSI D’ARTÈRIES CEREBRALS | CEREBROVASCULAR | ISCHEMIC STROKE |
| CIM10 | I63.4 | INFART CEREBRAL SECUNDARI A EMBÒLIA D’ARTÈRIES CEREBRALS | CEREBROVASCULAR | ISCHEMIC STROKE |
| CIM10 | I63.5 | INFART CEREBRAL SECUNDARI A OCLUSIÓ O ESTENOSI NO ESPECIFICADA D’ARTÈRIES CEREBRALS | CEREBROVASCULAR | ISCHEMIC STROKE |
| CIM10 | I63.6 | INFART CEREBRAL SECUNDARI A TROMBOSI DE VENES CEREBRALS, NO PIOGEN | CEREBROVASCULAR | ISCHEMIC STROKE |
| CIM10 | I63.8 | ALTRES INFARTS CEREBRALS | CEREBROVASCULAR | ISCHEMIC STROKE |
| CIM10 | I63.9 | INFART CEREBRAL, NO ESPECIFICAT | CEREBROVASCULAR | ISCHEMIC STROKE |
| CIM10 | I65 | OCLUSIÓ I ESTENOSI D’ARTÈRIES PRECEREBRALS SENSE OCASIONAR INFART CEREBRAL | PERIPHERAL | CAROTID/CEREBRAL DISEASE |
| CIM10 | I65.0 | OCLUSIÓ I ESTENOSI L’ARTÈRIA VERTEBRAL | PERIPHERAL | CAROTID/CEREBRAL DISEASE |
| CIM10 | I65.1 | OCLUSIÓ I ESTENOSI DE L’ARTÈRIA BASILAR | PERIPHERAL | CAROTID/CEREBRAL DISEASE |
| CIM10 | I65.2 | OCLUSIÓ I ESTENOSI DE L’ARTÈRIA CARÒTIDA | PERIPHERAL | CAROTID/CEREBRAL DISEASE |
| CIM10 | I65.3 | OCLUSIÓ I ESTENOSI MÚLTIPLE BILATERAL D’ARTÈRIES PRECEREBRALS | PERIPHERAL | CAROTID/CEREBRAL DISEASE |
| CIM10 | I65.8 | OCLUSIÓ I ESTENOSI D’ALTRES ARTÈRIES PRECEREBRALS | PERIPHERAL | CAROTID/CEREBRAL DISEASE |
| CIM10 | I65.9 | OCLUSIÓ I ESTENOSI D’UNA ARTÈRIA PRECEREBRAL NO ESPECIFICADA | PERIPHERAL | CAROTID/CEREBRAL DISEASE |
| CIM10 | I70.2 | ATEROSCLEROSI DE LES ARTÈRIES DELS MEMBRES | PERIPHERAL | PERIPHERAL ARTERIAL DISEASE (PAD) |
| CIM10 | I73.9 | MALALTIA VASCULAR PERIFÈRICA, NO ESPECIFICADA | PERIPHERAL | PERIPHERAL ARTERIAL DISEASE (PAD) |
| CIM9 | 6 | PROCEDIMENTS ENS VASOS SANGUINIS | PERIPHERAL | REVASCULARIZATION |
| CIM9 | 61 | ANGIOPLÀSTIA PERCUTÀNIA DE VAS -OS EXTRACRANIAL -S | PERIPHERAL | REVASCULARIZATION |
| CIM9 | 62 | ANGIPL PERCUTÀNIA O ATERECTO VAS INTRACEREBRAL | CEREBROVASCULAR | CEREBRAL REVASCULARIZATION |
| CIM9 | 360 | ELIMINACIÓ OBSTRUCCIÓ ARTÈRIA CORONÀRIA I INSERCIÓ DE STENT | CORONARY | CORONARY REVASCULARIZATION |
| CIM9 | 361 | ANASTOMOSI DE DESVIACIÓ PER A REVASCULARITZACIÓ CARDÍACA | CORONARY | CORONARY REVASCULARIZATION |
| CIM9 | 3610 | DESVIACIÓ AORTOCORONÀRIA P/A REVASCULARITZACIÓ CARDÍACA,NSP | CORONARY | CORONARY REVASCULARIZATION |
| CIM9 | 362 | REVASCULARITZACIÓ CARDÍACA P/IMPLANTACIÓ ARTERIAL;INDIRECTA | CORONARY | CORONARY REVASCULARIZATION |
| CIM9 | 363 | ALTRES REVASCULARITZACIONS CARDÍAQUES | CORONARY | CORONARY REVASCULARIZATION |
| CIM9 | 3639 | ALT.REVASCULARITZACIÓ CARDÍACA; ABRASIÓ EPICARDI | CORONARY | CORONARY REVASCULARIZATION |
| CIM9 | 3979 | ALTRES TIPUS DE PROCEDIMENTS EN ALTRES VASOS | PERIPHERAL | REVASCULARIZATION |
| CIM9 | 3990 | IMPL STENT NO ALLIBERADOR FÀRMACS VAS PERIFÈRIC NO CORONARI | PERIPHERAL | REVASCULARIZATION |
| CIM9 | 410 | INFART DE MIOCARDI AGUT | CORONARY | MIOCARDIAL INFARCTION |
| CIM9 | 4100 | INFART DE MIOCARDI AGUT DE PARET ANTEROLATERAL | CORONARY | MIOCARDIAL INFARCTION |
| CIM9 | 41000 | INFART DE MIOCARDI AGUT DE PARET ANTEROLATERAL, EPISODI NO ESPECIFICAT | CORONARY | MIOCARDIAL INFARCTION |
| CIM9 | 41001 | INFART DE MIOCARDI AGUT DE PARET ANTEROLATERAL, EPISODI INICIAL | CORONARY | MIOCARDIAL INFARCTION |
| CIM9 | 41002 | INFART DE MIOCARDI AGUT DE PARET ANTEROLATERAL, EPISODI POSTERIOR | CORONARY | MIOCARDIAL INFARCTION |
| CIM9 | 4101 | INFART AGUT MIOCARDI D’ALTRA PARET ANTERIOR | CORONARY | MIOCARDIAL INFARCTION |
| CIM9 | 41010 | IAM D’ALTRA PARET ANTERIOR, ASSISTÈNCIA INESPECIFICADA | CORONARY | MIOCARDIAL INFARCTION |
| CIM9 | 41011 | IAM D’ALTRA PARET ANTERIOR, ASSISTÈNCIA INICIAL | CORONARY | MIOCARDIAL INFARCTION |
| CIM9 | 41012 | IAM D’ALTRA PARET ANTERIOR, ASSISTÈNCIA SUBSEGÜENT | CORONARY | MIOCARDIAL INFARCTION |
| CIM9 | 4102 | INFART AGUT MIOCARDI, PARET INFEROLATERAL | CORONARY | MIOCARDIAL INFARCTION |
| CIM9 | 41020 | IAM, PARET INFEROLATERAL, ASSISTÈNCIA INESPECIFICADA | CORONARY | MIOCARDIAL INFARCTION |
| CIM9 | 41021 | IAM, PARET INFEROLATERAL, ASSISTÈNCIA INICIAL | CORONARY | MIOCARDIAL INFARCTION |
| CIM9 | 41022 | INFART DE MIOCARDI AGUT DE LA PARET INFEROLATERAL, EPISODI POSTERIOR | CORONARY | MIOCARDIAL INFARCTION |
| CIM9 | 4103 | INFART DE MIOCARDI AGUT DE LA PARET INFEROPOSTERIOR | CORONARY | MIOCARDIAL INFARCTION |
| CIM9 | 41030 | INFART DE MIOCARDI AGUT DE LA PARET INFEROPOSTERIOR, EPISODI NO ESPECIFICAT | CORONARY | MIOCARDIAL INFARCTION |
| CIM9 | 41031 | INFART DE MIOCARDI AGUT DE LA PARET INFEROPOSTERIOR, EPISODI INICIAL | CORONARY | MIOCARDIAL INFARCTION |
| CIM9 | 41032 | INFART DE MIOCARDI AGUT DE LA PARET INFEROPOSTERIOR, EPISODI POSTERIOR | CORONARY | MIOCARDIAL INFARCTION |
| CIM9 | 4104 | INFART DE MIOCARDI AGUT D’UNA ALTRA PART DE LA PARET INFERIOR | CORONARY | MIOCARDIAL INFARCTION |
| CIM9 | 41040 | INFART DE MIOCARDI AGUT D’UNA ALTRA PART DE LA PARET INFERIOR, EPISODI NO ESPECIFICAT | CORONARY | MIOCARDIAL INFARCTION |
| CIM9 | 41041 | INFART DE MIOCARDI AGUT D’UNA ALTRA PART DE LA PARET INFERIOR, EPISODI INICIAL | CORONARY | MIOCARDIAL INFARCTION |
| CIM9 | 41042 | INFART DE MIOCARDI AGUT D’UNA ALTRA PART DE LA PARET INFERIOR, EPISODI POSTERIOR | CORONARY | MIOCARDIAL INFARCTION |
| CIM9 | 4105 | INFART DE MIOCARDI AGUT D’UNA ALTRA PART DE LA PARET LATERAL | CORONARY | MIOCARDIAL INFARCTION |
| CIM9 | 41050 | INFART DE MIOCARDI AGUT D’UNA ALTRA PART DE LA PARET LATERAL, EPISODI NO ESPECIFICAT | CORONARY | MIOCARDIAL INFARCTION |
| CIM9 | 41051 | INFART DE MIOCARDI AGUT D’UNA ALTRA PART DE LA PARET LATERAL, EPISODI INICIAL | CORONARY | MIOCARDIAL INFARCTION |
| CIM9 | 41052 | INFART DE MIOCARDI AGUT D’UNA ALTRA PART DE LA PARET LATERAL, EPISODI POSTERIOR | CORONARY | MIOCARDIAL INFARCTION |
| CIM9 | 4106 | INFART DE MIOCARDI AGUT DE PARET POSTERIOR VERITABLE | CORONARY | MIOCARDIAL INFARCTION |
| CIM9 | 41060 | INFART DE MIOCARDI AGUT DE PARET POSTERIOR VERITABLE, EPISODI NO ESPECIFICAT | CORONARY | MIOCARDIAL INFARCTION |
| CIM9 | 41061 | INFART DE MIOCARDI AGUT DE PARET POSTERIOR VERITABLE, EPISODI INICIAL | CORONARY | MIOCARDIAL INFARCTION |
| CIM9 | 41062 | INFART DE MIOCARDI AGUT DE PARET POSTERIOR VERITABLE, EPISODI POSTERIOR | CORONARY | MIOCARDIAL INFARCTION |
| CIM9 | 4107 | INFART SUBENDOCARDÍAC AGUT | CORONARY | MIOCARDIAL INFARCTION |
| CIM9 | 41070 | INFART SUBENDOCARDÍAC AGUT, EPISODI NO ESPECIFICAT | CORONARY | MIOCARDIAL INFARCTION |
| CIM9 | 41071 | INFARTO SUBENDOCÁRDICO,EPISODIO DE ATENCIÓN INICIAL | CORONARY | MIOCARDIAL INFARCTION |
| CIM9 | 41072 | INFART SUBENDOCARDÍAC AGUT, EPISODI POSTERIOR | CORONARY | MIOCARDIAL INFARCTION |
| CIM9 | 4108 | INFART DE MIOCARDI AGUT D’ALTRES LOCALITZACIONS ESPECIFICADES | CORONARY | MIOCARDIAL INFARCTION |
| CIM9 | 41080 | INFART DE MIOCARDI AGUT D’ALTRES LOCALITZACIONS ESPECIFICADES, EPISODI NO ESPECIFICAT | CORONARY | MIOCARDIAL INFARCTION |
| CIM9 | 41081 | INFART DE MIOCARDI AGUT D’ALTRES LOCALITZACIONS ESPECIFICADES, EPISODI INICIAL | CORONARY | MIOCARDIAL INFARCTION |
| CIM9 | 41082 | INFART DE MIOCARDI AGUT D’ALTRES LOCALITZACIONS ESPECIFICADES, EPISODI POSTERIOR | CORONARY | MIOCARDIAL INFARCTION |
| CIM9 | 4109 | INFART DE MIOCARDI AGUT DE LOCALITZACIÓ NO ESPECIFICADA | CORONARY | MIOCARDIAL INFARCTION |
| CIM9 | 41090 | INFART DE MIOCARDI AGUT DE LOCALITZACIÓ NO ESPECIFICADA, EPISODI NO ESPECIFICAT | CORONARY | MIOCARDIAL INFARCTION |
| CIM9 | 41091 | INFART DE MIOCARDI AGUT DE LOCALITZACIÓ NO ESPECIFICADA, EPISODI INICIAL | CORONARY | MIOCARDIAL INFARCTION |
| CIM9 | 41092 | INFART DE MIOCARDI AGUT DE LOCALITZACIÓ NO ESPECIFICADA, EPISODI POSTERIOR | CORONARY | MIOCARDIAL INFARCTION |
| CIM9 | 411 | ALTRES FORMES AGUDES I SUBAGUDES DE CARDIOPATIA ISQUÈMICA | CORONARY | ANGOR |
| CIM9 | 4110 | SÍNDROME POSTINFART DE MIOCARDI | CORONARY | MIOCARDIAL INFARCTION |
| CIM9 | 4111 | SÍNDROME CORONÀRIA INTERMÈDIA | CORONARY | ANGOR |
| CIM9 | 4118 | ALTRES FORMES AGUDES I SUBAGUDES DE CARDIOPATIA ISQUÈMICA | CORONARY | ANGOR |
| CIM9 | 41181 | OCLUSIÓ CORONÀRIA AGUDA SENSE INFART DE MIOCARDI | CORONARY | ANGOR |
| CIM9 | 41189 | ALTRES FORMES AGUDES I SUBAGUDES DE CARDIOPATIA ISQUÈMICA | CORONARY | ANGOR |
| CIM9 | 412 | INFART ANTIC MIOCARDI; INFART MIOCARDI GUARIT DIAGNÒSTIC ECG | CORONARY | MIOCARDIAL INFARCTION |
| CIM9 | 413 | ANGINA DE PIT | CORONARY | ANGOR |
| CIM9 | 4130 | ANGINA DE DECÚBIT; ANGINA NOCTURNA | CORONARY | ANGOR |
| CIM9 | 4131 | ANGINA DE PRINZMETAL; ANGINA DE PIT VARIANT | CORONARY | INDETERMINATE CORONARY ARTERY DISEASE |
| CIM9 | 4139 | ALTRES TIPUS DÂ’ANGINA DE PIT I ANGINA DE PIT NO ESPECIFICADA | CORONARY | ANGOR |
| CIM9 | 414 | ALTRES FORMES DE CARDIOPATIA ISQUÈMICA CRÒNICA | CORONARY | INDETERMINATE CORONARY ARTERY DISEASE |
| CIM9 | 41402 | ATEROSCLEROSI CORONÀRIA D’EMPELT VENÓS AUTÒLEG | CORONARY | CORONARY REVASCULARIZATION |
| CIM9 | 41403 | ATEROSCLEROSI CORONÀRIA D’EMPELT BIOLÒGIC NO AUTÒLEG | CORONARY | CORONARY REVASCULARIZATION |
| CIM9 | 41404 | ATEROSCLEROSI CORONÀRIA D’EMPELT DE DERIVACIÓ ARTERIAL | CORONARY | CORONARY REVASCULARIZATION |
| CIM9 | 41405 | ATEROSCLEROSI CORONÀRIA D’EMPELT DERIVACIÓ INESPECIFICAT;NOS | CORONARY | CORONARY REVASCULARIZATION |
| CIM9 | 41407 | ATEROSCLEROSI EMPELT DERIVACIÓ (ARTERIAL,VENOSA) COR TRASPLA | CORONARY | CORONARY REVASCULARIZATION |
| CIM9 | 4148 | ALTRES FORMES ESPECIFICADES DE CARDIOPATIA ISQUÈMICA CRÒNICA | CORONARY | INDETERMINATE CORONARY ARTERY DISEASE |
| CIM9 | 4149 | CARDIOPATIA ISQUÈMICA CRÒNICA NO ESPECIFICADA | CORONARY | INDETERMINATE CORONARY ARTERY DISEASE |
| CIM10 | I50.1 | INSUFICIÈNCIA VENTRICULAR ESQUERRA | HEART FAILURE | HEART FAILURE_OTHERS |
| CIM10 | I50.9 | INSUFICIÈNCIA CARDÍACA, NO ESPECIFICADA | HEART FAILURE | HEART FAILURE_OTHERS |
| CIM10 | I50.90 | INSUFICIÈNCIA CARDÍACA, NO ESPECIFICADA | HEART FAILURE | HEART FAILURE_OTHERS |
| CIM10 | I50.91 | INSUFICIÈNCIA CARDÍACA, NO ESPECIFICADA | HEART FAILURE | HEART FAILURE_OTHERS |
| CIM9 | 428 | INSUFICIÈNCIA CARDÍACA | HEART FAILURE | HEART FAILURE_OTHERS |
| CIM9 | 4281 | INSUFICIÈNCIA CARDÍACA ESQUERRA; EDEMA AGUT DE PULMÓ | HEART FAILURE | HEART FAILURE_OTHERS |
| CIM9 | 4282 | INSUFICIÈNCIA CARDÍACA SISTÒLICA | HEART FAILURE | HEART FAILURE_OTHERS |
| CIM9 | 42820 | INSUFICIÈNCIA CARDÍACA SISTÒLICA INESPECIFICADA | HEART FAILURE | HEART FAILURE_OTHERS |
| CIM9 | 42822 | INSUFICIÈNCIA CARDÍACA SISTÒLICA CRÒNICA | HEART FAILURE | HEART FAILURE_OTHERS |
| CIM9 | 42823 | INSUFICIÈNCIA CARDÍACA SISTÒLICA CRÒNICA AGUDITZADA | HEART FAILURE | HEART FAILURE_OTHERS |
| CIM9 | 4283 | INSUFICIÈNCIA CARDÍACA DIASTÒLICA | HEART FAILURE | HEART FAILURE_OTHERS |
| CIM9 | 42830 | INSUFICIÈNCIA CARDÍACA DIASTÒLICA INESPECIFICADA | HEART FAILURE | HEART FAILURE_OTHERS |
| CIM9 | 42832 | INSUFICIÈNCIA CARDÍACA DIASTÒLICA CRÒNICA | HEART FAILURE | HEART FAILURE_OTHERS |
| CIM9 | 42833 | INSUFICIÈNCIA CARDÍACA DIASTÒLICA CRÒNICA AGUDITZADA | HEART FAILURE | HEART FAILURE_OTHERS |
| CIM9 | 4284 | INSUFICIÈNCIA CARDÍACA COMBINADA, SISTÒLICA I DIASTÒLICA | HEART FAILURE | HEART FAILURE_OTHERS |
| CIM9 | 42840 | INSUFICIÈNCIA CARDÍACA COMBINADA SISTÒLICA/DIASTÒLICA INESP. | HEART FAILURE | HEART FAILURE_OTHERS |
| CIM9 | 42842 | INSUFICIÈNCIA CARDÍACA COMBINADA SISTÒLICA/DIASTÒLICA CRÒNIC | HEART FAILURE | HEART FAILURE_OTHERS |
| CIM9 | 42843 | INSUFICIÈNCIA CARDÍACA COMBINADA SISTÒ./DIASTÒ.CRÒN.AGUDITZ. | HEART FAILURE | HEART FAILURE_OTHERS |
| CIM9 | 4289 | INSUFICIÈNCIA CARDÍACA INESP.; NOS: CARDÍACA, MIOCARDÍACA | HEART FAILURE | HEART FAILURE_OTHERS |
| CIM9 | 4297 | DETERMINADES SEQÜELES D’INFART DE MIOCARDI NO CLASSIFICADES A CAP ALTRE LLOC | CORONARY | MIOCARDIAL INFARCTION |
| CIM9 | 42971 | DEFECTE SEPTAL CARDÍAC ADQUIRIT POSTERIOR A INFART DE MIOCARDI | CORONARY | MIOCARDIAL INFARCTION |
| CIM9 | 42979 | ALTRES SEQÜELES D’INFART DE MIOCARDI NO CLASSIFICADES A CAP ALTRE LLOC | CORONARY | MIOCARDIAL INFARCTION |
| CIM9 | 431 | HEMORRÀGIA INTRACEREBRAL | CEREBROVASCULAR | HEMORRHAGIC STROKE |
| CIM9 | 432 | ALTRES HEMORRÀGIES INTRACRANIALS I HEMORRÀGIES INTRACRANIALS NO ESPECIFICADES | CEREBROVASCULAR | HEMORRHAGIC STROKE |
| CIM9 | 433 | OCLUSIÓ I ESTENOSI D’ARTÈRIES PRECEREBRALS | PERIPHERAL | CAROTID/CEREBRAL DISEASE |
| CIM9 | 4330 | OCLUSIÓ I ESTENOSI ARTÈRIA BASILAR | PERIPHERAL | CAROTID/CEREBRAL DISEASE |
| CIM9 | 43300 | OCLUSIÓ I ESTENOSI ARTÈRIA BASILAR, SENSE INFART CEREBRAL | PERIPHERAL | CAROTID/CEREBRAL DISEASE |
| CIM9 | 43301 | OCLUSIÓ I ESTENOSI ARTÈRIA BASILAR, AMB INFART CEREBRAL | CEREBROVASCULAR | ISCHEMIC STROKE |
| CIM9 | 4331 | OCLUSIÓ I ESTENOSI ARTÈRIA CARÒTIDE | PERIPHERAL | CAROTID/CEREBRAL DISEASE |
| CIM9 | 43310 | OCLUSIÓ I ESTENOSI ARTÈRIA CARÒTIDE, SENSE INFART CEREBRAL | PERIPHERAL | CAROTID/CEREBRAL DISEASE |
| CIM9 | 43311 | OCLUSIÓ I ESTENOSI ARTÈRIA CARÒTIDE, AMB INFART CEREBRAL | CEREBROVASCULAR | ISCHEMIC STROKE |
| CIM9 | 4332 | OCLUSIÓ I ESTENOSI ARTÈRIA VERTEBRAL | PERIPHERAL | CAROTID/CEREBRAL DISEASE |
| CIM9 | 43320 | OCLUSIÓ I ESTENOSI ARTÈRIA VERTEBRAL, SENSE INFART CEREBRAL | PERIPHERAL | CAROTID/CEREBRAL DISEASE |
| CIM9 | 43321 | OCLUSIÓ I ESTENOSI ARTÈRIA VERTEBRAL, AMB INFART CEREBRAL | CEREBROVASCULAR | ISCHEMIC STROKE |
| CIM9 | 4333 | OCLUSIÓ I ESTENOSI MÚLTIPLE/BILATERAL ARTÈRIES PRECEREBRALS | PERIPHERAL | CAROTID/CEREBRAL DISEASE |
| CIM9 | 43330 | OCLUSIÓ/ESTENOSI MÚLT./BILAT.ART.PRECEREBRALS,S/INFART CERV. | PERIPHERAL | CAROTID/CEREBRAL DISEASE |
| CIM9 | 43331 | OCLUSIÓ/ESTENOSI MÚLT./BILAT.ART.PRECEREBRALS,A/INFART CERV. | CEREBROVASCULAR | ISCHEMIC STROKE |
| CIM9 | 4338 | OCLUSIÓ I ESTENOSI ALTR.ARTÈRIES PRECEREBRALS ESPECIFICADES | PERIPHERAL | CAROTID/CEREBRAL DISEASE |
| CIM9 | 43380 | OCLUSIÓ/ESTENOSI ALT.ART.PRECEREBRALS ESP.,S/INFART CERVELL | PERIPHERAL | CAROTID/CEREBRAL DISEASE |
| CIM9 | 43381 | OCLUSIÓ/ESTENOSI ALT.ART.PRECEREBRALS ESP.,A/INFART CERVELL | CEREBROVASCULAR | ISCHEMIC STROKE |
| CIM9 | 4339 | OCLUSIÓ I ESTENOSI ARTÈRIA PRECEREBRAL INESP.; NOS | PERIPHERAL | CAROTID/CEREBRAL DISEASE |
| CIM9 | 43390 | OCLUSIÓ/ESTENOSI ARTÈRIA PRECEREBRAL INESP.,S/INFART CERVELL | PERIPHERAL | CAROTID/CEREBRAL DISEASE |
| CIM9 | 43391 | OCLUSIÓ/ESTENOSI ARTÈRIA PRECEREBRAL INESP.,A/INFART CERVELL | CEREBROVASCULAR | ISCHEMIC STROKE |
| CIM9 | 434 | OCLUSIÓ D’ARTÈRIES CEREBRALS | PERIPHERAL | CAROTID/CEREBRAL DISEASE |
| CIM9 | 4340 | TROMBOSI CEREBRAL; TROMBOSI D’ARTÈRIES CEREBRALS | CEREBROVASCULAR | ISCHEMIC STROKE |
| CIM9 | 43400 | TROMBOSI CEREBRAL SENSE MENCIÓ D’INFART CEREBRAL | CEREBROVASCULAR | ISCHEMIC STROKE |
| CIM9 | 43401 | TROMBOSI CEREBRAL AMB INFART CEREBRAL | CEREBROVASCULAR | ISCHEMIC STROKE |
| CIM9 | 4341 | EMBOLISME CEREBRAL | CEREBROVASCULAR | ISCHEMIC STROKE |
| CIM9 | 43410 | EMBOLISME CEREBRAL, SENSE INFART CEREBRAL | CEREBROVASCULAR | ISCHEMIC STROKE |
| CIM9 | 43411 | EMBOLISME CEREBRAL, AMB INFART CEREBRAL | CEREBROVASCULAR | ISCHEMIC STROKE |
| CIM9 | 4349 | OCLUSIÓ ARTÈRIA CEREBRAL INESPECIFICADA | CEREBROVASCULAR | ISCHEMIC STROKE |
| CIM9 | 43490 | OCLUSIÓ D’ARTÈRIA CEREBRAL NO ESPECIFICADA SENSE MENCIÓ D’INFART CEREBRAL | CEREBROVASCULAR | ISCHEMIC STROKE |
| CIM9 | 43491 | OCLUSIÓ D’ARTÈRIA CEREBRAL NO ESPECIFICADA AMB INFART CEREBRAL | CEREBROVASCULAR | ISCHEMIC STROKE |
| CIM9 | 435 | ISQUÈMIA CEREBRAL TRANSITÒRIA | CEREBROVASCULAR | TRANSIENT ISCHEMIC ATTACK (TIA) |
| CIM9 | 4350 | SÍNDROME DE L’ARTÈRIA BASILAR | CEREBROVASCULAR | TRANSIENT ISCHEMIC ATTACK (TIA) |
| CIM9 | 4351 | SÍNDROME DE L’ARTÈRIA VERTEBRAL | CEREBROVASCULAR | TRANSIENT ISCHEMIC ATTACK (TIA) |
| CIM9 | 4352 | SÍNDROME DEL FURT DE LA SUBCLÀVIA | CEREBROVASCULAR | TRANSIENT ISCHEMIC ATTACK (TIA) |
| CIM9 | 4353 | SÍNDROME DE L’ARTÈRIA VERTEBROBASILAR | CEREBROVASCULAR | TRANSIENT ISCHEMIC ATTACK (TIA) |
| CIM9 | 4358 | ALTRES ISQUÈMIES CEREBRALS TRANSITÒRIES ESPECIFICADES | CEREBROVASCULAR | TRANSIENT ISCHEMIC ATTACK (TIA) |
| CIM9 | 4359 | ISQUÈMIA CEREBRAL TRANSITÒRIA NO ESPECIFICADA | CEREBROVASCULAR | TRANSIENT ISCHEMIC ATTACK (TIA) |
| CIM9 | 436 | MALALTIA CEREBROVASCULAR AGUDA MAL DEFINIDA; APOPLEXIA | CEREBROVASCULAR | ISCHEMIC STROKE |
| CIM9 | 4377 | AMNÈSIA GLOBAL TRANSITÒRIA | CEREBROVASCULAR | TRANSIENT ISCHEMIC ATTACK (TIA) |
| CIM9 | 44021 | ATEROSCLEROSI D’EXTREMITATS AMB CLAUDICACIÓ INTERMITENT | PERIPHERAL | PERIPHERAL ARTERIAL DISEASE (PAD) |
| CIM9 | 44022 | ATEROSCLEROSI D’EXTREMITATS AMB DOLOR DE REPÒS | PERIPHERAL | PERIPHERAL ARTERIAL DISEASE (PAD) |
| CIM9 | 44023 | ATEROSCLEROSI D’EXTREMITATS AMB ULCERACIÓ | PERIPHERAL | PERIPHERAL ARTERIAL DISEASE (PAD) |
| CIM9 | 44024 | ATEROSCLEROSI D’EXTREMITATS AMB GANGRENA | PERIPHERAL | PERIPHERAL ARTERIAL DISEASE (PAD) |
| CIM9 | 4403 | ATEROSCLEROSI EN EMPELT DE DERIVACIÓ DE LES EXTREMITATS | PERIPHERAL | REVASCULARIZATION |
| CIM9 | 44030 | ATEROSCLEROSI D’EMPELT DE DERIVACIÓ DE LES EXTREMITATS NO ESPECIFICAT | PERIPHERAL | REVASCULARIZATION |
| CIM9 | 44031 | ATEROSCLEROSI D’EMPELT DE DERIVACIÓ DE LES EXTREMITATS VENÓS AUTÒLEG | PERIPHERAL | REVASCULARIZATION |
| CIM9 | 44032 | ATEROSCLEROSI D’EMPELT DE DERIVACIÓ DE LES EXTREMITATS BIOLÒGIC NO AUTÒLEG | PERIPHERAL | REVASCULARIZATION |
| CIM9 | 4404 | OCLUSIÓ TOTAL CRÒNICA D’ARTÈRIA DE LES EXTREMITATS | PERIPHERAL | PERIPHERAL ARTERIAL DISEASE (PAD) |
| CIM9 | 4439 | MAL.VASCULAR PERIFÈRICA INESP.; CLAUDICACIÓ INTERMITENT | PERIPHERAL | PERIPHERAL ARTERIAL DISEASE (PAD) |
| CIM9 | V4581 | ESTAT DE DERIVACIÓ AORTOCORONÀRIA | CORONARY | CORONARY REVASCULARIZATION |
| CIM9 | V4582 | ESTAT D’ANGIOPLÀSTIA CORONÀRIA TRANSLUMINAL PERCUTÀNIA | CORONARY | CORONARY REVASCULARIZATION |

|  | **No event (n=190,599)** | **Event (n=57,152)** | **p** |
| --- | --- | --- | --- |
| **Sex F/M (n, (%))** | 94,820 (49.7)/95,779 (50.3) | 25585 (44.8)/31,567 (55.2) | <0.01 |
| **Age (years)** | 65.6 ±11.9 | 71.1±10.5 | <0.01 |
| **Deprivation index (Q5)** | 29,347 (21.7) | 8,367 (21.9) | 0.225 |
| **Smoking (n, (%)** |  | |  |
| No | 105,941 (64.7) | 31,249 (62.0) | <0.01 |
| Former | 39,370 (18.4) | 9,389 (18.6) |  |
| Current | 27,886 (17.0) | 9.736 (19.3) |  |
| **Type 2 diabetes duration (years)** | 6.0 ±5.0 | 7.1± 5.7 | <0.01 |
| **Type 2 diabetes therapy (n, (%))** |  | |  |
| No treatment | 55,322 (29.0) | 11,484 (20.1) | <0.01 |
| OHA monotherapy | 66,694 (35.0) | 17,725 (31.0) |  |
| OHA combined | 42,648 (22.4) | 14,821 (25.9) |  |
| OHA plus insulin | 17,298 (9.1) | 8,717 (15.3) |  |
| Insulin without OHA | 8,637 (4.5) | 4,405 (7.7) |  |
| **HbA1c (%)** | 6.8 ±1.5 | 7.0±1.6 | <0.01 |
| **BMI (Kg/m^2^)** | 30.2±5.0 | 30.3±5.2 | 0.531 |
| **Hypertension (n (%))** | 115,549 (60.6) | 41,418 (72.5) | 0.959 |
| **SBP (mmHg)** | 135 (15.2) | 139 (16.7) | <0.01 |
| **DBP (mmHg)** | 77.2 (9.28) | 75.7 (9.82) | <0.01 |
| **Hypotensive treatment (n (%))** | 118,345 (62.1) | 44,079 (77.1) | <0.01 |
| **Hyperlipidemia (n,%)** | 117,763 (59.0) | 37,149 (65.0) | <0.01 |
| **Total cholesterol (mg/dl)** | 198 (38.5) | 195 (39.7) | <0.01 |
| **HDL-cholesterol (mg/dl)** | 50.4 (13.0) | 49.5 (13.0) | <0.01 |
| **Triglycerides>150 mg/dl (n,(%)** | 47,656 (39.6) | 15,410 (40.8) | 0.012 |
| **LDL-cholesterol (mg/dl)** | 118 (32.6) | 116 (33.2) | <0.01 |
| **Remnant-cholesterol (mg/dl)** | 28.6 (13.4) | 29.1 (13.4) | <0.01 |
| **Statin use (n (%))** | 84,260 (44.2) | 28,645 (50.1) | <0.01 |
| **eGFR <60 ml/min (n (%))** | 18,879 (14.9) | 10,693 (26.7) | <0.01 |
| **UAE>30 mg/gr (n (%))** | 7,720 (12.8) | 4,600 (23.7) | <0.01 |
| **Any microvascular complication (n (%))** | 17,717 (9.3) | 10.129 (17.7) | <0.01 |

**Supplementary Table 3:** Clinical baseline characteristics of participants with or without a cardiovascular event during follow-up.

Data are expressed as mean ± standard deviation, median [25^th^ percentile-75^th^ percentile], or number of participants (n) (%).

F/M: female/male; BMI: body mass index; SBP: systolic blood pressure; DBP: diastolic blood pressure: HDL-cholesterol: high-density lipoprotein cholesterol; LDL-cholesterol: low-ddensity-lipoprotein cholesterol; eGFR: estimated glomerular filtration rate; UAE: urinary albumin excretion

**Supplementary Figure 1:**

**
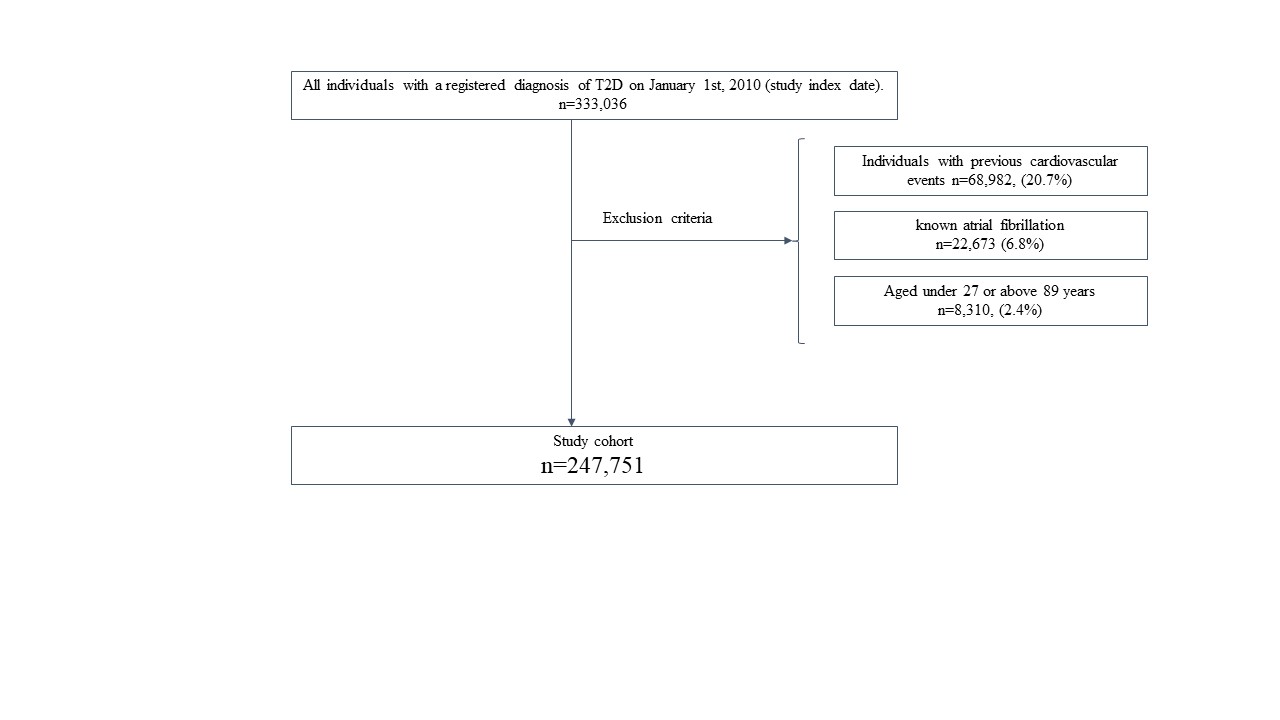
**

**Supplementary Figure 2**: Relative contribution, in percentage, of specific cardiovascular diseases to the first CVD event in men and women for the entire cohort

**Supplementary Figure 3**: Forrest plot illustrating the impact of different cardiovascular risk factors on the relative risk of a cardiovascular event in men (blue) and women (red)


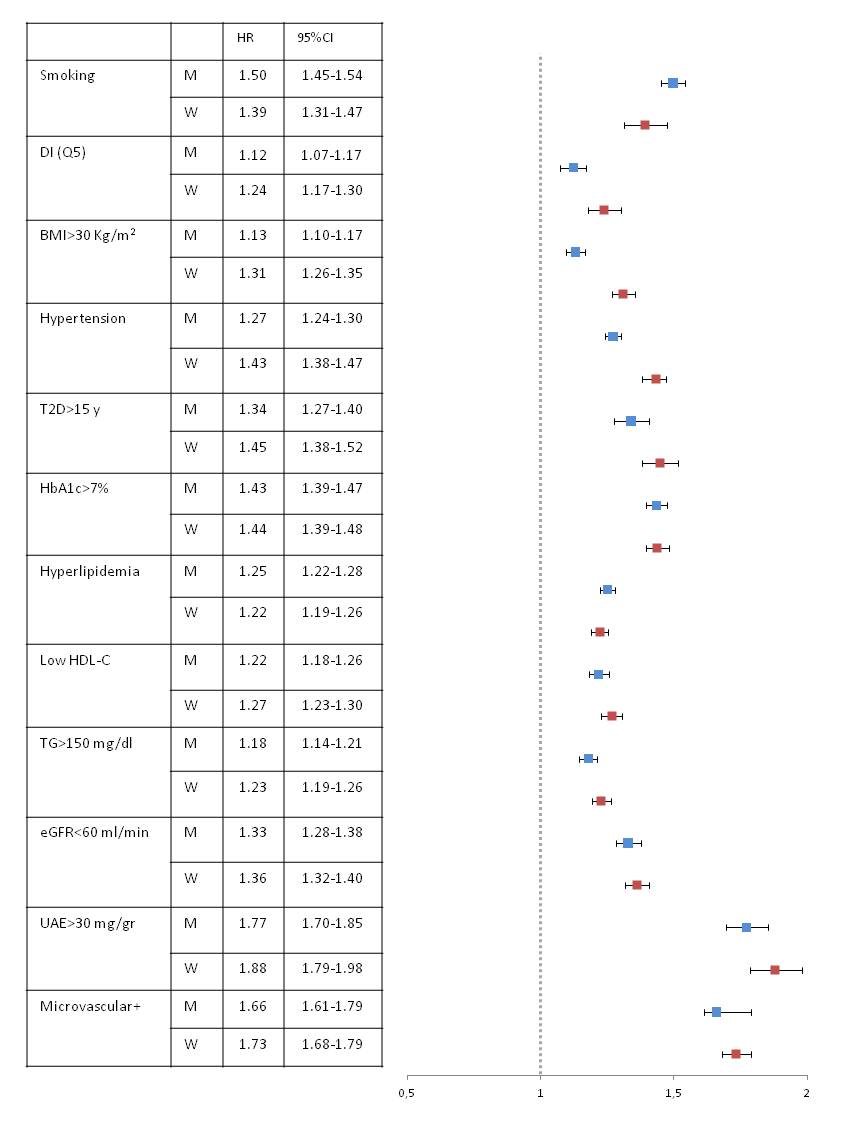


HR: hazard ratio; 95%CI: 95% confidence interval.

**Supplementary Figure 4**: Forrest plot illustrating the impact of different cardiovascular risk factors on the relative risk of a cardiovascular event in the pre-specified age groups.


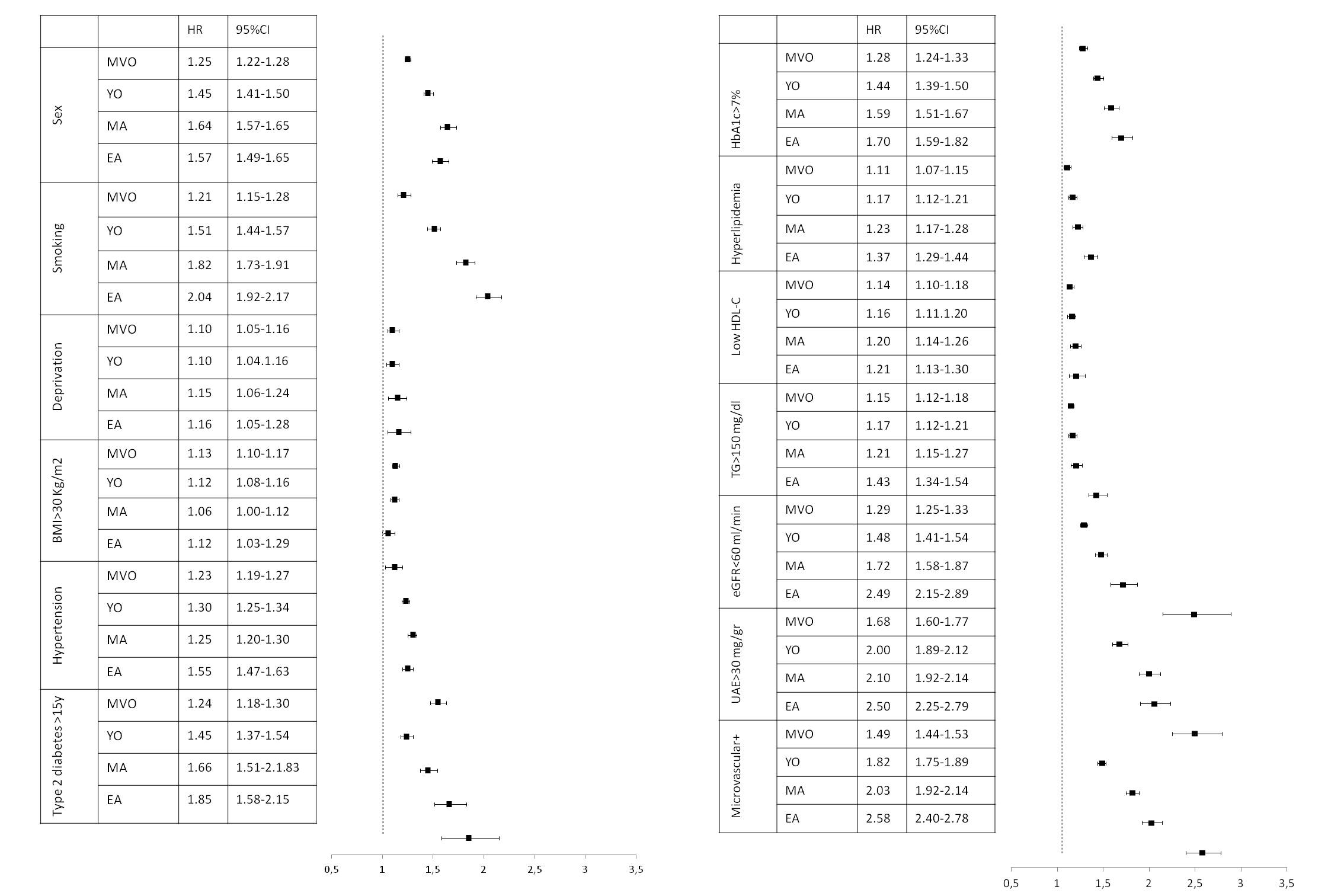


HR: Hazard ratio; 95% CI: 95% confidence interval. MVO: middle to very old; YO: young old; MA: middle-aged; EA: early adulthood. Given the low number of events among the young age category, the hazard ratios for cardiovascular risk factors for this age-category were not included in the figure.
